# Genetic therapies for neurological diseases

**DOI:** 10.1016/j.pharmr.2025.100093

**Published:** 2025-03-10

**Authors:** Ahad A. Rahim, Manju A. Kurian, Haiyan Zhou, Ross Ferguson, Sarah J. Tabrizi, Gabriele Lignani, Kristian Aquilina, Simon N. Waddington

**Affiliations:** 1 Department of Pharmacology, School of Pharmacy, University College London, London, United Kingdom; 2 Developmental Neurosciences, Zayed Centre for Research into Rare Disease in Children, Great Ormond Street Institute of Child Health, University College London, London, United Kingdom; 3 Department of Neurology, Great Ormond Street Hospital for Children, London, United Kingdom; 4 Genetics and Genomic Medicine Research and Teaching Department, Great Ormond Street Institute of Child Health, University College London, London, United Kingdom; 5 National Institute for Health and Care Research, Great Ormond Street Hospital Biomedical Research Center, London, United Kingdom; 6 Huntington’s Disease Centre, Department of Neurodegenerative Disease, UCL Queen Square Institute of Neurology, University College London, London, United Kingdom; 7 Dementia Research Institute at University College London, London, United Kingdom; 8 Research Department of Epilepsy, Queen Square Institute of Neurology, University College London, London, United Kingdom; 9 Department of Neurosurgery, Great Ormond Street Hospital, London, United Kingdom; 10 Department of Maternal and Fetal Medicine, Elizabeth Garrett Anderson Institute for Women’s Health, University College London, London, United Kingdom

## Abstract

Often, gene therapy reviews concentrate upon specific therapeutic modalities—particularly either viral vector-mediated or a nonviral approach. Here, we draw together a comprehensive array of knowledge across the field of genetic therapy for genetic neurological disease. The sections on preclinical and clinical application of viral vectors are followed by sections on RNA-based therapies and then by antisense oligonucleotide approaches also in preclinical and clinical settings. We present a separate section on gene editing strategies and conclude with a section elaborating on the neurosurgical techniques and the expertise required for clinical application of many of these technologies.

**Significance Statement:**

Genetic therapies have significant potential to treat life-limiting neurological diseases. This review examines the different approaches, clinical successes, and considerations on how to deploy them.

## I Overarching introduction

Genetic therapies for neurological diseases represent a rapidly advancing field focused on correcting or compensating for faulty genes responsible for a range of debilitating brain and nervous system disorders. These therapies include DNA- or RNA-based approaches to replace, silence, or edit the genetic content of the cell. Although theoretically simple, on a practical level this is challenging as cells of the body have defense mechanisms preventing external genetic material from entering and eliminating it once it has entered. Therefore, the effectiveness of these approaches is dependent on efficient delivery systems to transport genetic cargo into cells. These can broadly be categorized into either viral or nonviral delivery systems. Furthermore, these must be combined with advanced surgical techniques to overcome the physical encapsulation of the brain within the skull or the highly selective blood–brain barrier (BBB). Challenges remain, including immune responses, off-target effects, and ensuring long-term expression and safety.

Despite the numerous hurdles that require to be overcome, technological advances and learnings from preclinical and in-human studies, numerous products have now achieved market approval, and hundreds are in clinical trials.

In this review, we aim to provide a comprehensive overview of genetic therapies for neurological disease; this comprises viral vector technologies, RNA therapeutics, antisense oligonucleotide (ASO) therapies, and gene editing. We explore preclinical and clinical successes and obstacles and conclude with a section discussing surgical delivery of all these modalities.

## II Viral vectors for neurological genetic therapies

### A Introduction

The concept of genetic therapies is delivery of polynucleic acids to cells of the body for therapeutic benefit. Depending on the type of therapeutic nucleic acid, this process varies in efficiency. A delivery system or “vector” is required to carry nucleic acid into the cell. It is generally accepted that the more efficient these vectors are, the better the therapy will be.

Over at least 500 million years,^1^ viruses have evolved to successfully deliver their genetic cargo into cells. Therefore, decades of research into the optimal vector has used viruses. The type of viral vector to use is complex and dependent on (1) the disease indication, (2) the size of the genetic cargo, (3) the duration of expression required, (4) the type of cell or organ being targeted, (5) manufacturability, and (6) safety. This section will look at some examples of viral vectors that have been developed and deployed for use in treating neurological diseases.

### B Adeno-associated viral vectors

Adeno-associated viruses (AAVs) are parvoviruses that were first discovered in the 1960s as contaminants in adenoviral preparations.^2^ They are composed of a ∼4.7 kb single-stranded DNA genome encapsulated within an icosahedral capsid made up of 3 proteins. The relatively small size of AAV means that as a gene therapy vector, the maximum genetic cargo they can accommodate is ∼4.5 kb. The archetypal virus integrates into the human genome specifically at chromosome site 19q13.4^3^^,^^4^; however, AAV vector, devoid of requisite integration machinery, remains predominantly episomal and instead integrates randomly at a low frequency.^5^^,^^6^ AAV vectors have become the most used genetic therapy vector for neurological diseases with a number of clinical trials in progress.^7^ With more than 100 natural variants and many serotypes, there is a diverse toolbox that binds different cellular receptors and exhibits different biodistribution profiles. AAV2 shows less spread following administration into the brain and is well suited for indications where targeted delivery is beneficial such as dopamine transporter deficiency syndrome^8^ and now licensed Upstaza (PTC Therapeutics) for the treatment of aromatic L-amino acid decarboxylase (AADC) deficiency (AADCd).^9^ Global brain diseases that affect multiple and broad anatomical regions of the large and complex brain are challenging targets.^10^ AAV9 has a broad biodistribution and been used in preclinical studies for a range of neurological diseases that display global brain pathology including neurodegenerative lysosomal storage diseases,^7^ spastic paraplegias,^11^^,^^12^ epilepsies,^13^ and neurodevelopmental conditions^14^^,^^15^ among many others.

AAV vectors can be administered into the central nervous system (CNS) via different routes of administration including intraparenchymal, intracerebroventricular (ICV), intracisterna magna, and intrathecal (IT) injections. The ability of AAV9 to cross the BBB and deliver genes to the brain and spine was a major step forward in noninvasively delivering gene therapy via an intravenous injection for neurological diseases such as licensed Zolgensma (Novartis Gene Therapies) for the treatment of spinal muscular atrophy (SMA).^16^ Although the success of intravenously administered AAV9 for SMA can be accredited to biodistribution that is well suited to this condition and high transduction of motor neurons in the spinal cord, this route of administration is not suitable or efficient enough for all neurological conditions.

AAV vectors are now being developed through engineering of capsids that allow for targeting of specific cell types, enhanced transduction profile, or the ability to cross the BBB. This has been largely achieved through rational design or directed evolution approaches.^17^ Rational design relies on knowledge of capsid stability or the capsid-cell receptor interaction and making targeted improvements. This has identified capsids that have increased biodistribution^18^ or better BBB penetrance.^19^ Directed evolution approaches are higher throughput and depend on the generation of high complexity capsid libraries. These can be generated through recombination and shuffling of capsid sequences, peptide insertions into capsid coding regions or error-prone polymerase chain reaction. In vitro or in vivo screening of these libraries under selective pressure has identified a number of novel capsids exhibiting neural cell specificity,^20^ enhanced BBB penetrance^21^, ^22^, ^23^ or enhanced expression.^24^

The presence of pre-existing neutralizing antibodies to AAV is a challenge, and seropositive patient could be ineligible for treatment. The prevalence of neutralizing antibodies is linked to age and earlier intervention may reduce the risk.^25^ Furthermore, direct administration into the cerebrospinal fluid (CSF) may allow for some tolerance of pre-existing neutralizing antibodies.^26^

As discussed in subsequent sections of this review, naturally occurring capsids are used in successfully licensed gene therapies. Other neurological diseases that are more challenging (eg, require higher levels of transduction, broader biodistribution or lowering of doses) would benefit from the deployment of new capsid variants such as those described above. AAV9 is a popular choice for evaluating gene therapies that require broad biodistribution due to multiple parts of the brain being affected. However, biodistribution in nonhuman primate (NHP) studies following delivery to the CSF highlights delivery of genetic material is not homogeneous. Some regions of the brain are better transduced than others and that deeper structures in the brain are less transduced.^27^ Furthermore, contrast agent and positron emission tomography studies in NHPs also highlight that a significant proportion of the administered vector into the CSF escapes to the periphery.^27^^,^^28^ More effective and efficient vectors will enhance biodistribution and lower doses required, which would improve safety profiles, reduce the burden and cost of manufacturing, and therefore potentially reduce the cost of AAV-based genetic therapies. However, moving new AAVs into the clinic is complex and requires thorough evaluation. Most importantly, are these new vectors manufacturable at scale and at clinical grade, is their efficacy and efficiency in animal models maintained in a human, and are they safe. It is important to note that aspects of biodistribution of an AAV vector are linked to the route and mode of administration. This is expanded upon later in this review in the context of surgical approaches for administering genetic therapy vectors.

### C Retroviral/lentiviral vectors

Retroviruses are single-stranded RNA viruses that can enter dividing cells, convert their genome to double-stranded DNA using reverse transcriptase, and then integrate into the host genome. Work on developing retroviral vectors dates to the 1980s.^29^ The capacity to deliver a genetic cargo of 8 kb and the ability to integrate it into the host genome has made them attractive vectors for applications where sustained gene expression is desirable or where the targets cell is rapidly dividing. The limitation of gamma-retroviral vectors to only deliver their genetic cargo to dividing cells led to development of the closely related lentiviral vectors isolated from different species.^30^^,^^31^ These vectors allowed for postmitotic cells such as neurons of the brain to become amenable to efficient transduction and delivery of genes.^32^^,^^33^ Further investigation also provided evidence of a different and safer profile of integration compared with their gamma-retroviral counterparts.^34^ In combination with the insertion of self-inactivating elements, lentiviral vectors have been generally considered the safer vector.

Both gamma-retroviral and lentiviral vectors use glycoproteins on their surface envelope to interact with cell receptors for internalization. The tropism of vectors can be modulated through changing the envelope and glycoproteins. Numerous pseudotypes have been used to either broaden or narrow tropisms in the CNS. Pseudotyping with the rabies virus envelope confers retrograde transport from the neuronal synapse to the cell body.^35^^,^^36^ This potentially provides access to the CNS via a noninvasive intramuscular injection.

Lentiviral vectors administered directly into the CNS have been used extensively in preclinical studies of gene therapy for various neurological indications in mice or larger animal models. The most advanced program has been developed for Parkinson’s disease (PD) (ProSavin) using a lentiviral vector based on the equine infectious anemia virus that is administered directly into the brain parenchyma and is discussed further in this review. Direct injection into the CSF for maximum biodistribution has been limited, especially when compared with AAV vectors. This is not entirely unexpected given that the concentrations of lentiviral or gamma-retroviral viral particles that can be achieved per unit volume are orders of magnitude lower than AAV. The risk of insertional mutagenesis observed in clinical trials using gamma-retroviral vectors for immunodeficiencies^37^^,^^38^ has slowed research involving direct in vivo administration of lentiviral vectors. Integration defective lentiviral vectors were specifically developed for short-term expression in dividing cells or where integration is not required for long-term expression such as nondividing neurons.^39^ The introduction of mutations in the integrase gene renders the vector unable to integrate into the host cell genome and instead exists as an episome. This provides a safer strategy where the risk of insertional mutagenesis is diminished. Integration defective lentiviral vectors using different pseudotypes have been tested in the brain of mice and shown to successfully mediate neuronal transduction.^40^

The use of lentiviral vectors in hematopoietic stem cell gene therapy for neurological diseases holds significant potential. The rationale is based on lentiviral-corrected hematopoietic stem cells differentiating into cells of myeloid lineage that can cross the BBB and cross-correct neighboring cells such as neurons, astrocytes, and oligodendrocytes. This led to successful preclinical and clinical studies in conditions such as leukodystrophies^41^^,^^42^ and mucopolysaccharidosis (MPS),^43^^,^^44^ and recently the breakthrough lentiviral-based licensed drug Libmeldy (Orchard Therapeutics) for the treatment of metachromatic leukodystrophy. However, as cross-correction is a vital component of this approach, it is restricted to gene products that are soluble and secreted from the transduced cells.

### D Canine adenovirus vectors

Adenoviruses consist of a nonenveloped icosahedral protein shell of ∼95 nm, enclosing a linear double-stranded genome, which can range from ∼25 to 48 kb. Human adenoviruses belong to the *Mastadenovirus* genus and are grouped into 7 species, defined by blood serotype and more recently genetic phylogeny.^45^ Adenovirus serotype 5 of the C species has been the most widely used and studied. Adenovirus serotype 5 and other human serotypes, and hybrids thereof, have been used for gene transfer to vascular smooth muscle cells^46^ and for oncolytic virotherapy.^47^ There were several early studies using these for gene delivery to mouse^48^ and rat brain.^49^^,^^50^ However, antivector immune responses^51^^,^^52^ curtailed broader exploitation.

Nevertheless, redemption came in 1997, when Klonjkowski et al^53^ commenced development of a vector from canine adenovirus serotype 2 (CAV-2). This vector demonstrates exquisite tropism for neurons^54^ because CAV-2 binds to, and is internalized with, the coxsackievirus and adenovirus receptor.^55^ Coxsackievirus and adenovirus receptor is highly expressed in the brains of rodents^55^ (particularly maturing and mature neurons^56^) and in the primate *Microcebus murinus*.^57^ CAV-2 has also been used to deliver, effectively, to cynomolgus and rhesus macaques.^58^ Early generation vectors were created by deleting the E1 and or E3 regions of the viral genome to provide a 7-kb transgene payload capacity. Later, helper-dependent CAV-2 vectors, devoid of all viral genome except for flanking inverted terminal repeats, have permitted a transgene payload capacity of ∼36 kb.^59^

Neurotropism has been exploited in numerous rodent studies investigating neural circuits, neuromotor, and neurocognitive behavior (reviewed and listed in the study by Del Rio et al^60^). These include, among many, identification of 2 distinct dopaminergic pathway circuits,^61^ the mechanism by which visual perception drives adaptive threat response,^62^ the integration of predatory motor function by the central nucleus of the amygdala,^63^ prefrontal cortex control of reward-seeking behavior,^64^ selective routing of sensory information by prefrontal cortex dopamine,^65^ and the modulation of feeding behavior by cholinergic signaling of the basal forebrain.^66^

### E Other viral vectors

Other viral vectors have been tested for their ability and potential to deliver gene therapy to the brain. Adenoviruses have a double-stranded DNA genome and third-generation helper-dependent (gutless) vectors, which contain none of the original virus genome except for 5′ and 3′ inverted terminal repeats and packaging signal, can accommodate ≈37 kb of genetic cargo.^67^ Despite various studies demonstrating gene delivery to the brain using human and canine variants, their progress as gene therapy vectors has not progressed for clinical use.^68^^,^^69^ Concerns around immunogenicity have hampered progress although oncolytic adenoviral vectors are in clinical trials for glioblastoma and gliosarcoma.^70^ Herpes simplex virus carry a double-stranded DNA genome and vectors have the capacity to accommodate ∼30 kb of genetic cargo.^67^ Historically, the use of this vector in the CNS has been challenging due to concerns around cytotoxicity and immunogenicity associated to the inclusion of immediate-early sequences.^71^ However, newer iterations of the herpes simplex virus vectors that have these sequences removed are showing promise for longer-term expression in the brain.^72^

## III Clinical translation of viral gene therapy: successes, challenges, and prospects for future development

### A Introduction

Therapeutic targeting of the brain presents unique challenges for viral gene therapy. Potential hurdles relate to penetration of the skull, crossing the BBB, and achieving either accurate regional delivery or widespread brain biodistribution.^73^ Nevertheless, viral vector-mediated gene therapies have now been trialed in a broad spectrum of neurological disorders presenting clinically from early infancy to late adulthood. A broad range of AAV and lentiviral vectors have been used in the clinic, delivered through various intravenous, intra-CSF, and intraparenchymal routes.^7^

### B Autologous stem cell approaches

Approaches using ex vivo gene transfer for autologous stem cell gene therapy have been trialed for several neurological disorders. Cerebral adrenoleukodystrophy (ALD) is an X-linked inborn error of metabolism caused by mutations in *ABCD1*, leading to ALD protein deficiency. Without therapeutic intervention, the natural history of disease results in childhood motor regression, cognitive decline, and death. Elivaldogene tavalentivec (Lenti-D or Skysona, Genetix Biotherapeutics) has now been approved for use in cerebral ALD, where infusion of autologous CD34+ cells transduced with Lenti-D has shown significant benefit in clinical trial.^41^ Lentivirus vector is used to deliver functional coding sequence for ATP-binding cassette, subfamily D, member 1 (ABCD1) to the stem cells. This ex vivo gene therapy strategy has been shown to be significantly disease-modifying, with almost 90% survival in the trial cohort. Importantly, treated boys were reported to have no major functional disability, with minimal clinical symptoms. Autologous stem cell gene therapy approaches have also been applied to metachromatic leukodystrophy, where the use of atidarsagene autotemcel (arsa-cel) has also shown significant clinical benefit, especially in children who were treated before symptom onset.^42^ Most treated patients either gained developmental motor skills within the expected range for healthy children or showed stabilization of their motor abilities, retaining the ability to ambulate. Moreover, most had normal cognitive development, with either prevention or delay of central and peripheral demyelination and brain atrophy. Similar approaches have also been undertaken for the lysosomal storage disorders, MPSIIIA, and MPSI-Hurler variant.^74^^,^^75^ Despite successes in the field, ex vivo gene therapy presents several challenges related to immunogenicity, risk of lentivirus-related insertional mutagenesis, and complications from transplantation. The use of autologous stem cell gene therapy is also limited to inborn errors of metabolism where such cross-correction is possible. Furthermore, this approach may also not be ideal for neurological conditions where time-sensitive treatments within a therapeutic window are needed, as processes related to ex vivo cell transduction, expansion, and engraftment can often take several months. Technological advances that speed us these processes will be important in future therapeutic development.

### C Direct in vivo gene therapy

Systemic gene therapy may be needed for multisystemic diseases that affect other body organs as well as the brain. Systemic delivery has been trialed for complex inborn errors of metabolism, albeit with variable results. For example, despite success using a systemic gene therapy approach for MPSIIIA in animal models,^76^ clinical trials were terminated due to reported lack of efficacy, lack of drug supply, and commercial barriers. Although systemic approaches present significant advantages (by providing safer, less invasive viral gene delivery for multisystem diseases), better vector transduction efficacy will be necessary to optimize delivery to the brain. It also highlights that although some diseases like SMA are well suited to systemic delivery, others are not. This could be linked with whether the biodistribution is appropriate to the condition, the vector’s tropism to a particular cell type which is critical to rescue for the disease, whether neurons outside the brain are the major target and taking into account that much of the vector will sequester in the liver following intravenous injection.

Both isolated and combined intra-CSF delivery approaches have been used in viral gene therapy clinical trials. IT administration of viral gene therapy via lumbar puncture has been used for several neurogenetic disorders. In autosomal recessive giant axonal neuropathy, an IT-delivered AAV9-based approach has resulted in preservation of motor function with alteration of the natural history of disease.^77^ IT delivery has also been trialed for other inborn errors of metabolism (IEM) and neurodegenerative diseases including GM2 gangliosidosis type 1 (Tay-Sachs disease), Batten’s disease (CLN3, CLN6, and CLN7), type 2 Sandhoff disease, and Alzheimer’s disease (AD), with delivery of neuroprotective APOE2. ICV delivery combined with intravenous delivery has been trialed for a patient with Canavan’s disease, with this combination aiming to increase both brain and systemic gene transduction. Two years after treatment, there was an increase in white matter myelination, improvement in motor function, and absence of the typically severe epilepsy.^78^ For Tay-Sachs disease, caused by mutation of the hexosaminidase A (HEXA) gene, a combination of IT with intracisterna magna HEXA gene delivery led to seizure-freedom 2.5 years after therapy in 1 child. In another infant, although combining IT with intrathalamic delivery led to clinical stabilization in the short term, long-term efficacy was not achieved, with disease progression and evolution of seizures by 2 years of age.^79^ Increased CSF HEXA activity was evident in both these patients.

Intraparenchymal brain gene delivery has recently provided highly encouraging results through AMT-130, the first disease modifying treatment for HD which has recently been reported by uniQure.^80^ It comprises an AAV5 vector carrying an engineered microRNA targeting the huntingtin gene transcript for degradation and is delivered directly to the striatum by MRI-guided stereotaxic infusion. Unlike many prior mutant huntingtin targeting approaches, it is also able to target an alternate transcript which encodes a shorter toxic isoform of huntingtin and is a major driver of pathology.^81^ The phase I/II trial participants received either high or low doses and the topline data showed significant and meaningful slowing of disease progression in the high dose cohort at thirty-six months after administration in comparison to external controls. This was seen in two key HD progression scores, a 75% slowing by composite Unified Huntington’s Disease Rating Scale (cUHDRS) and 60% slowing by total functional capacity (TCF), as well as reduced CSF NfL. Treatment was also reported to be well tolerated with adverse effects primarily associated with surgical delivery. The success seen so far with AMT-130 offers great excitement for the treatment of HD, both for AMT-130 as a therapeutic in itself but also in terms of the success of the target, platform and delivery for future approaches.

Intraparenchymal brain gene delivery has also been trialed in patients with different forms of MPSs, Canavan disease, CLN2, and AD.^82^, ^83^, ^84^, ^85^, ^86^ Viral gene therapy injected as an AAV2 vector into multiple brain regions through stereotactic targeting was deemed to be safe but with limited efficacy overall, likely attributed to insufficiently widespread vector biodistribution in the brain.

Targeted delivery of viral gene therapy into specific parenchymal regions of the brain is useful for treating conditions where the region of primary pathology is well defined, such as PD with neurodegeneration of the nigrostriatal pathway. Here, stereotactic targeting of the putamen has been used for viral vector-mediated gene delivery of a broad range of transgenes, including AADC (either in isolation or in combination with tyrosine hydroxylase and guanosine triphosphate cyclohydrolase 1, ProSavin)^87^, ^88^, ^89^ as well as glial cell line–derived neurotrophic factor, and glutamic acid decarboxylase, and neurturin.^90^, ^91^, ^92^, ^93^

Delivery of the transgene DDC increases dopamine by providing the key enzyme that catalyzes the conversion of levodopa to dopamine; in clinical trial, this approach was initially deemed to be safe, and high dose gene therapy cohorts showed therapeutic benefit with improved motor function, reduced medication burden, and quality of life. However, the ongoing randomized, double-blind, sham-controlled phase 2 clinical trial with higher infusion volumes was recently placed on hold, with brain magnetic resonance imaging (MRI) abnormalities observed. In therapeutic trials of ProSavin, combining AADC enzyme with other components of the dopamine synthesis pathway was safe and well tolerated in patients with PD, with moderate improvements in motor symptoms over baseline reported in the majority of patients, evaluated up to 5 years of follow-up. Supplementation of glutamic acid decarboxylase through subthalamic delivery, aimed at enhancing inhibitory GABAergic signaling, has also shown motor benefit for treated individuals.^91^ Similarly trials in glial cell line–derived neurotrophic factor and neurturin viral gene therapy have shown safety^92^ and modest efficacy,^93^ respectively, particularly for patients treated earlier in their disease course. This indeed might be one of the major challenges in optimizing viral gene therapy for PD, where treatment is likely to be time-sensitive, before irreparable striatonigral neurodegeneration has taken place.

Although many of these PD trials have had, at best, modest results, more impressive clinical benefit has been seen with targeted putaminal delivery of the DDC transgene for AADCd.^94^, ^95^, ^96^, ^97^, ^98^ AADCd is a rare inherited primary neurotransmitter disorder resulting from biallelic variants in *DDC*, encoding the final enzyme that catalyzes the conversion of 5-hydroxytryptophan to serotonin and L-dopa to dopamine. Affected children have a resultant deficiency of dopamine, epinephrine and norepinephrine, and serotonin, and classically present with severe neurodevelopmental delay, hypotonia, oculogyric crises, and life-threatening autonomic features. Many children with untreated AADCd will never achieve full head control. Eladocagene exuparvovec (Upstaza) is an AAV2-based viral vector delivered to bilateral putamina via stereotactic neurosurgical delivery. Clinical trials have shown that many patients have major improvements in cognitive and motor function, with significant developmental gains. Furthermore, many AADCd patients had less severe and frequent oculogyric crises associated with improved mood, seating, temperature, and dopamine production. Upstaza received regulatory approval for the treatment of AADCd in the UK and Europe in 2022. Patients with AADCd have also been treated with an alternative midbrain gene therapy approach, which takes advantage of anterograde axonal transport mechanisms to enhance vector distribution to key dopaminergic networks within the nigrostriatal, mesocortical, and mesolimbic pathways. A clinical trial focusing on targeted midbrain delivery to the substantia nigra and ventral tegmental area (with image-guided convection-enhanced delivery [CED] to optimize vector distribution within this region) showed safety and impressive efficacy; treated children showed significant motor gains, cognitive development, reduction, or cessation of oculogyric crises, with increase in brain dopamine levels.^99^ It would be ideal to compare midbrain versus putaminal injections but small patient numbers would make this challenging. Future challenges for AADCd relate to addressing the issue of targeting the serotonergic system which is currently not targeted via the available midbrain and putaminal delivery strategies.

Although the use of oncolytic viral therapy for the treatment of childhood and adult gliomas falls outside the remit of this review, we must acknowledge the application of these vectors to the brain. These have predominantly focused on herpes simplex virus and adenoviral vector platforms and have been reviewed by others.^100^^,^^101^

## IV RNA-based therapies for neurological genetic disorders

### A Introduction

RNA therapeutics are a class of medications that use RNA-based molecules to regulate target gene expression for intervention in various medical conditions. These therapies offer advantages over conventional drug strategies, such as small molecules or antibodies, as they can address druggable targets at the DNA, RNA, and protein levels. In addition, RNA-based therapies provide therapeutic benefits by modulating disease-associated targets in a more gene-specific manner, offering a tailored approach to treatment.

**Fig. 1:**
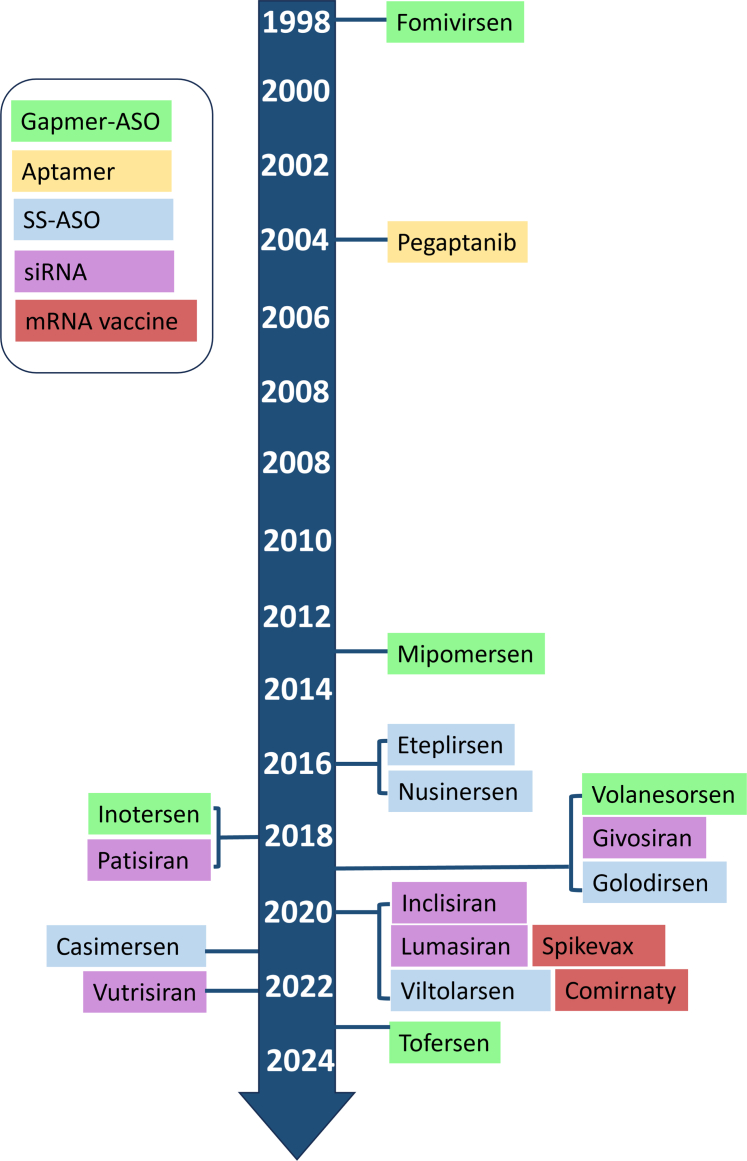
A timeline of regulatory approval for disease-modifying RNA drugs. ss-ASO, splice-switching antisense oligonucleotide.

RNA therapeutics, like many other technologies, has undergone distinct phases of development, transitioning from initial enthusiasm in the 1970s,^102^ to periods of stagnation and even abandonment, and then to a revival, as demonstrated by the recent surge in regulatory approvals of RNA-based drugs for various conditions,^103^^,^^104^ and as pivotal components in COVID vaccines. Since the approval of the first ASO drug, fomivirsen, to treat cytomegalovirus retinitis in 1998, over 16 disease-modifying RNA drugs having received regulatory approval from the Food and Drug Administration (FDA) or the European Medicines Agency (Fig. 1). These approvals catalyzed the development of new RNA drugs, using various modalities (eg, ASO, small-interfering RNA [siRNA], and mRNA) chemistries (eg, phosphorothioate 2′-O-methoxyethyl [MOE] and phosphorodiamidate morpholino oligomers [PMOs]), and delivery conjugations (eg, lipid nanoparticle [LNP] packaging and N-acetylgalactosamine [GalNAc] conjugation). These are described in greater detail in relevant sections below. Moreover, the COVID-19 pandemic played a significant role in promoting the development and application of mRNA vaccines, which has reignited interest in RNA therapeutics.

The landscape of RNA-based therapeutics has seen a remarkable expansion in recent years. This is driven by advancements in chemical modifications, novel conjugation technologies, and the identification of new disease targets, attributed to the advanced genetic studies and deeper insights into the associated molecular mechanisms. The progress has spawned a diverse array of RNA-based approaches within this therapeutic class. Among these are ASO, aptamers, siRNA, microRNA (miRNA), small activating RNA (saRNA), mRNA, and CRISPR-mediated genome or RNA editing. Each approach operates through distinct mechanism of action and offers unique clinical applications, collectively regulating target gene expression at the DNA (eg, CRISPR), RNA (eg, ASO, siRNA, saRNA, or miRNA) or protein (eg, mRNA and gapmer) levels. This multifaceted toolkit holds promise for addressing a wide spectrum of diseases, fueled by the versatility and precision of RNA-based interventions. Among the various modalities, ASOs constitute of the majority of RNA therapeutics in the preclinical and clinical development. Considering its paramount importance and wide clinical applications in many disease areas, we will discuss about ASO therapeutics in a separate section in this review.

siRNA is a double-stranded short RNA molecule that can interfere with the expression of specific genes by targeting and degrading the corresponding mRNA via the RNA-induced silencing complex. siRNA therapeutics are designed to silence or downregulate the expression of disease-related genes and have thrived in recent years in metabolic disorders where liver is the target organ, benefited from new chemical modification in nucleotides, and various packaging and conjugation technologies.^105^ Successful examples include patisiran in LNP packaging and vutrisiran in GalNAc conjugation for hereditary transthyretin amyloidosis, givosiran for acute hepatic porphyria, lumasiran for hydroxy acid oxidase 1, inclisiran for hypercholesterolemia, or mixed dyslipidemia.^106^

Although there have been no approved siRNA drugs to directly target genes within the CNS, extensive preclinical proof-of-concept studies in cellular and animal models have demonstrated the therapeutic effects of siRNAs in neurological conditions. Potential targets include siRNAs to silence huntingtin (HTT) mRNA via generic^107^ or allele-specific silencing,^108^ or target its modifiers^109^^,^^110^ in Huntington’s disease (HD); to silence superoxide dismutase 1 (SOD1)^111^ or C9orf72^112^ in amyotrophic lateral sclerosis (ALS); to target BACE1,^113^ tau or amyloid precursor protein (APP),^114^ or apolipoprotein E^115^ for AD.^116^ Early phase clinical trials of siRNA are currently ongoing in neurological conditions. ALN-APP (NCT05231785), using siRNA trial to silence APP for early-onset AD, has showed promising interim results in clinical safety and efficacy.^117^ ALN-HTT02, using Alnylam’s proprietary C16-siRNA delivery system to downregulate HTT, has just started.

saRNA is another double-stranded short RNA molecule that can enhance target gene expression by recruiting the RNA-induced transcriptional activation complex to low-copy promoter-associated RNA, leading to transcriptional activation of the proximal gene.^118^ saRNA presents completely opposite biological functions to siRNA although they share similar structures and chemical components. saRNA can be delivered in vivo by LNPs, dendrimers, lipid, and polymer hybrids and aptamers.^119^ Although there has been no saRNA drug approved by regulatory, clinical trials of saRNAs have been studied in liver cancer with promising results.^120^ saRNA-mediated gene activation in CNS has also been explored for its potential in treating neurological conditions. Foxg1 gene expression in neural cells has been induced by saRNA in neonate mice as a prospective saRNA therapy of Rett syndrome.^121^ Although promising, challenges remain in the clinical translation of saRNA therapy in neurological conditions, where improved in vivo efficacy and novel efficient delivery systems in CNS are the key obstacles to tackle.^122^

miRNAs are small RNA molecules that play a role in posttranscriptional gene regulation. In miRNA therapeutics, synthetic miRNA mimics (also known as agomir) or inhibitors (also known as anti-miRs or antagomirs) are used to modulate the activity of endogenous miRNAs, influencing target gene expression. miRNA therapeutics is compelling in regulating gene expression in human cells and animal models. Despite extensive investigations in preclinical research, this therapeutics remains in its early development stage with only a few progressing to clinical translation and several facing termination due to lack of efficacy and toxicity issues. Most of the clinical trials are targeting cancer and fibrosis, although there has not been any miRNA drug approved by regulators.^123^, ^124^, ^125^ Clinical trials of miRNA are also underway in neurological conditions.^126^ AMT-130 (NCT04120493) is AAV5-delivered artificial miRNA targeting HTT gene,^127^^,^^128^ and has showed promising clinical outcomes in an early phase trial of HD.

mRNA therapeutics use the inherent cellular processes of mRNA transcription and translation to produce target protein and elicit the desired therapeutic effect by either replacing dysfunctional proteins or activating the immune system.^129^ Synthetic mRNAs are engineered in vitro to resemble natural mRNA while incorporated with modifications in structure design and nucleotide chemistry to improve stability and translational efficiency. Boosted by the success of mRNA vaccines during COVID-19 pandemic, the in vitro transcribed mRNA has now gained more interest as a therapeutic avenue for different disease conditions. Numerous preclinical studies have demonstrated the therapeutic potential of in vitro transcribed mRNA as a nonviral gene replacement approach for loss-of-function diseases. Despite the promise in preclinical studies, delivering therapeutic mRNA to target organs and cells remains a crucial challenge, especially to meet the demand of long-term gene replacement and to establish it as a general therapeutic modality with broad application to more disease conditions.^130^ Although no mRNA drugs have been approved as disease-modifying therapy, except as vaccines for viruses and cancers, this technology has presented promising potential in preclinical studies and clinical trials in metabolic diseases by targeting the liver.^131^^,^^132^ Preclinical studies of mRNA therapy have also emerged in neurological monogenic loss-of-function conditions,^133^ such LNP-encapsulated frataxin mRNA for Friedreich’s ataxia.^134^ However, the application of mRNA therapy in neurological diseases currently is still limited due to the challenges such as delivery in the CNS, frequent dosing, and potential immunogenicity and toxicity where more studies are still needed.

RNA aptamers are short, single-stranded RNA molecules that fold into unique 3-dimensional structures capable of specifically binding to a wide range of targets, including proteins, small molecules, nucleic acids, and even cells. RNA aptamers are typically generated through a process known as systematic evolution of ligands by exponential enrichment, wherein RNA libraries are iteratively screened and enriched for sequences that exhibit binding affinity to the desired target. Aptamers can also be engineered into aptamer-drug conjugates and targeted drug delivery systems, enabling their translation into therapeutic application.^135^ Pegaptanib is the only aptamer received approval for macular degeneration by inhibiting vascular endothelial growth factor activity to reduce pathological angiogenesis.^136^ The therapeutic potential of aptamers has been studied in cells and mouse models of neurological conditions, including aptamers against amyloid *β* or BACE1 for AD by interrupting the amyloidogenic pathway, and DNA aptamers for PD by targeting *α*-synuclein (SNCA).^137^ To overcome the delivery hurdle from BBB, a dual aptamer system comprising a transferrin receptor (TfR) aptamer and a circular tau aptamer (TfR-tau aptamer) presented the potential for the tauopathies.^138^ Although aptamers hold promise in eliminating pathological proteins in the CNS, their use in treating neurological diseases is still in the early stages of development.

CRISPR Cas-based therapeutics harness RNA molecules (guide RNAs) to direct the Cas enzymes to specific DNA or RNA sequences for targeted genome or RNA editing. This cutting-edge approach is categorized under RNA-based therapeutics owing to its reliance on RNA guides and targets. As a recent addition and one of the fast-growing RNA-based therapeutics, the CRISPR landscape is rapidly evolving. Given its extensive studies, diverse applications and rapid progress, ASO and CRISPR gene editing will be discussed separately in more detail in subsequent sections.

### B Successes and areas for improvement

The resurgence of RNA therapeutics is catalyzed by breakthroughs in several key areas: (1) novel RNA chemistry modifications—these modifications enhance stability, prolong the half-life, increase the bioavailability of therapeutic RNA molecules, and improve the pharmacokinetic properties of RNA therapeutics, making them more effective in clinical settings; (2) the evolution of genomic medicine and genetic diagnosis—this advancement has revolutionized our understanding of diseases at the molecular and genetic level, and unveiled novel targets for therapeutic intervention, allowing for more precise and personized treatment approaches^139^^,^^140^; (3) the field has seen significant progress in the development of novel RNA-based approaches on targeting different genetic defects; and (4) innovative delivery systems have been developed to enhance the biodistribution and overall efficacy of RNA therapeutics. These advancements have widened the scope of RNA-based therapeutics and increased their potential for clinical transition.

Like many emerging technologies in medicine, the field of RNA-based therapeutics has had to surmount several challenges during its maturation process. Scale-up manufacturing of synthetic nucleotides with reduced cost has been effectively tackled. The development of various chemical modifications to nucleotides has enhanced their resistance to metabolism by nucleases and improved their pharmacokinetic profiles. These fundamental breakthroughs have played a pivotal role in facilitating the clinical translation of numerous RNA therapeutics, as evidenced by the growing number of regulator-approved RNA drugs in recent years.

Despite the considerable progress, further improvement in RNA therapeutics is necessary to broaden their application across a wider range of disease areas. A major challenge in neurological disorders is to achieve efficient and targeted delivery of RNA therapeutics to the CNS. Currently, local administration via repeated IT or ICV injections is still the primary route of treatment. Efforts to enhance RNA molecule penetration across the BBB and target specific cell types in the CNS are actively under investigation.^141^^,^^142^ Various bioconjugations strategies have been used to promote intracellular uptake, enhance the biodistribution, and reduce clearance from circulation. These include polymers, lipids (eg, cholesterol, palmitoyl, and PEGylated lipid),^143^ peptides (eg, cell penetrating peptides [CPPs]),^144^ receptor ligands (eg, GalNAc) or antibodies, exosomes, and aptamers.^145^ Several brain-targeting peptides have been engineered and demonstrated potential in crossing the BBB, such as PMO nucleic acid internalization peptide (Pip) conjugates,^146^ TfR antibody with ASO conjugates,^147^ and exosome engineered with rabies virus glycoprotein.^148^ Although promising in preclinical studies, further investigations into their clinical efficacy and safety are needed before these modalities can be applied to patients. Given the ASOs make up a significant proportion of studies reaching the clinic, with notable successes but also failures, we have specifically discussed the challenges and progression in delivery to the CNS in the section below.

RNA therapeutics is not only applicable to common genetic disorders but also holds great promise for treating rare diseases, making it a cornerstone of personalized medicine. The advancement in genomic medicine and genetic diagnosis has paved the way for personalized genetic therapy, enabling the development of RNA therapeutics tailored to address the specific genetic causes. A notable example is milasen, an ASO therapy designed to correct a mutation identified in a single patient with Batten’s disease,^149^ showcasing the potential of RNA therapeutics as an individualized treatment approach for rare neurological conditions, and ushering in a new era of personalized medicine. To extend the benefits of individualized RNA therapeutics to more patients with rare diseases, international collaboration such as the global N=1 Collaborative (https://www.n1collaborative.org), the European collaboration 1M1M (https://www.1mutation1medicine.eu), and the UK Platform of Nucleic Acid Therapeutics Node (https://rd-research.org.uk) are underway to establish frame-works and standardize procedures for rapid translation from bench to bedside. Despite challenging, RNA therapeutics for neurological conditions represent a rapidly evolving field with the potential to revolutionize the treatment of various disorders affecting the nervous system.

## V Antisense oligonucleotides as therapeutics

### A Introduction

**Fig. 2:**
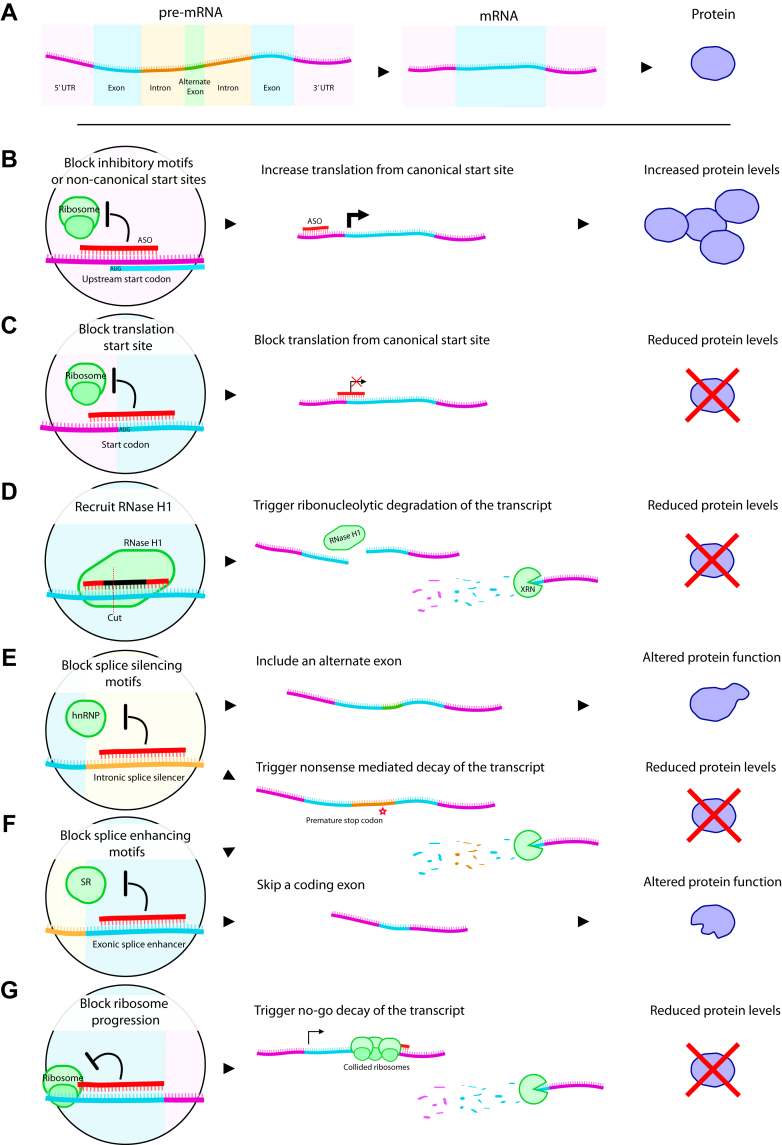
A summary of mechanisms through which ASOs can affect gene expression. (A) Transcription of a gene results initially in a pre-mRNA transcript which are comprised of a 5′ and 3′ untranslated regions (UTRs) flanked protein coding sequence in exons interspersed with noncoding introns and alternate or cryptic exons. Introns and unwanted exons are removed by the splicing machinery (mostly cotranscriptionally) to generate the mature protein coding transcript which goes on to be translated. ASOs can perturb multiple steps in this process to affect protein levels and function. (B) ASOs which bind and occupy inhibitory motifs or alternate upstream open reading frames in a transcript block their unproductive translation and can increase canonical translation of the transcript. (C) Similarly, an ASO occupying sites proximal to the canonical translational start site (the AUG codon) can block translation and lower protein levels. (D) Gapmer ASOs with a 5–10 bp DNA core recruit RNase H1, an endoribonuclease detector of DNA:RNA double-strand hybrids. RNase H1 cleaves the transcript which is then degraded further by XRN exoribonucleases. ASO hybridization to motifs in the transcript that direct the spliceosome can affect how the transcript is processed, altering the coding sequence of the mature transcript, stability and protein function; (E) Alternate exons or intronic sequences can be retained due to ASOs occupying splice silencing motifs blocking recruitment of factors such as hnRNP. (F) Similarly, exons can be selectively excluded by ASOs occupying splice enhancer motifs, preventing the binding of spliceosome factors such as serine-rich family proteins (SR). Where skipping or including sequence results in frameshifts and premature stop codons, the transcript undergoes nuclease dependent NMD reducing protein levels. (G) Degradation by the alternate no-go decay pathway can be triggered primarily by ASOs which bind toward the end of the transcript. These block ribosome progression and collisions recruit and activate no-go decay factors, again resulting in reduced protein levels.

ASOs are chemically synthesized sequences composed of 12–30 nucleotides designed to bind a specific RNA target by Watson-Crick base pairing. The first use of synthetic DNA oligonucleotides to inhibit translation was reported in the late 70s,^102^^,^^150^ almost 20 years before RNA interference was described.^151^^,^^152^ Over 40 years later, advances in ASO design have enabled quite fine manipulation of an RNA target including targeted degradation, stabilization, altered posttranscriptional RNA processing, and altered translation of protein coding transcripts (Fig. 2A). Which of these mechanisms is invoked is governed by the design of the ASO, its chemistry, the binding site to which it hybridizes, and the function of the target RNA. The ability to induce these effects through recruiting ribonucleases to the transcript, or by competing with other RNA-binding factors for regulatory motifs, makes ASOs a versatile tool with which to approach the pathophysiology of many brain diseases as both therapeutics and research tools.

### B Mechanism of action

Lowering expression of a target protein is a common therapeutic approach. This can be achieved through multiple mechanisms using ASOs, most commonly through targeted degradation of the transcript by ribonucleases (Fig.2D). The endoribonuclease RNase H1 is able to specifically detect and initiate degradation of the RNA strand of DNA:RNA heteroduplexes and many ASOs exploit this effect.^153^ In contrast to RNA interference–mediated degradation where the siRNA needs to be loaded into the RNA-induced silencing complex, these ASOs bind their target mRNA first which results in recruitment and activation RNase H1. RNase H1 is localized to both the nucleus and cytoplasm, as such RNase H1 recruitment to heteroduplexes can occur in both compartments. Degradation of cytoplasmic RNAs occurs more rapidly than nuclear retained RNAs in an RNase H1 concentration–dependent fashion.^154^, ^155^, ^156^ After the initial endonucleolytic cleavage of the duplex by RNase H1, further 5′–3′ exonucleases of the XRN family degrade the target RNA–XRN1 in the cytoplasm and XRN2 in the nucleus.^157^^,^^158^ These mechanisms occur rapidly—where ASOs are introduced into cells by transfection, reductions in mRNA levels can be observed as soon as 30 minutes later.^156^

In addition to the RNase H1–dependent mechanism, ASO design can also lead to altered protein levels simply by preventing the binding and function of RNA metabolic factors through steric blocking at key motifs in the transcript. Steric blocking ASOs have been able to block translation initiation when targeted to either the start codon or the cap formation site^159^; conversely many 5′- targeting strategies have been able to increase target protein levels through binding to regulatory motifs such as inhibitory elements or upstream open reading frames (Fig. 2B), increasing translation of the transcript.^160^, ^161^, ^162^ Although these 5′-targeting ASOs can either directly block or enhance translation of the transcript, ASOs designed to hybridize to the 3′ of the coding sequence can lower mature mRNA levels by triggering the no-go decay pathway. This occurs where translation is impeded by an obstacle, resulting in the collision of multiple ribosomes (Fig. 2G).^163^ The application of modalities such as these is entirely dependent on the pre-existing regulatory elements within the transcript.

Splice modulating ASOs or splice switching oligos (SSOs) are an important class of ASOs that also function through steric blocking in the nucleus. Splice switching can be exploited to retain expression of the encoded protein sequence but alter its function by favoring the translation of alternately spliced isoforms (Fig. 2, E and F). Alternate splicing allows for multiple protein variants to be generated from the same pre-mRNA and the choice of splice site usage depends on splice enhancers and silencers within the transcript, and the repertoire of RNA-binding protein complexes that interact with them.^164^ These interactions can be disrupted by ASOs designed to occupy motifs in the transcript, consequently altering how the spliceosome uses or ignores certain splice sites.

SSOs can drive inclusion of an exon by hybridizing to splice silencing sites, blocking recruitment of splice repressors (Fig. 2E) and can also drive exon exclusion from a transcript by instead targeting splice enhancer sequences (Fig. 2F). These 2 strategies are used by 2 different clinically approved ASOs in the treatment of SMA and Duchenne’s muscular dystrophy (DMD). In SMA, nusinersen drives inclusion of an otherwise skipped exon in the *SMN2* transcript which is then translated to an isoform able to partly compensate for the loss of SMN1 protein. In contrast, exon-skipping SSOs are a core focus of several ASO-based approaches for treating DMD.^165^ Here, exon skipping in the dystrophin transcript excludes exons carrying deletions, duplications, or point mutations and allows for translation of a partly functional dystrophin protein, similar to that found in the milder Becker’s muscular dystrophy.^166^

Finally, SSOs can also be designed to lower transcript levels by exploiting the nonsense-mediated decay (NMD) pathway (Fig. 2, E and F). Changes to the canonical splicing program of a target mRNA can lead to the retention of an intron, inclusion of a cryptic exon, or exclusion of a key exon. Where this results in changes to the reading frame or coding sequence of the transcript, premature stop codons may be introduced. These novel stop codons are recognized by the NMD factors triggering degradation of transcript by the exoribonuclease XRN family proteins.^167^

### C Current antisense oligonucleotides in clinical and preclinical development

In 2016, nusinersen was the first ASO to be approved to treat SMA by IT delivery in pediatric and adult patients. It is a fully phosphorothioate (PS) and MOE modified 18mer ASO.^168^^,^^169^ The majority of ASOs currently in trials for CNS disorders are either RNase H1–recruiting gapmers or splice modulators. As outlined previously, nusinersen acts to promote inclusion of the normally excluded exon 7 in the *SMN2* transcript by binding and occluding an intronic splice silencing motif. This results in an *SMN2* transcript that encodes an isoform able to compensate for the lack of SMN1.

Since the approval of nusinersen 8 years ago, aside from compassionate use and N-of-1 trials, only tofersen has been approved for the treatment of SOD1 ALS in the CNS.^170^ Tofersen is also delivered intrathecally and targets the SOD1 transcript. It was given accelerated approval after showing its ability to lower SOD1 and neurofilament light in blood and CSF.^171^ It is a gapmer with a mixed PS backbone and a central core of 10 DNA nucleotides flanked by 5 2′-MOE modified ribonucleotides allowing it to recruit RNase H1 on hybridization with SOD1 transcripts.^172^

ALS is a genetically complex disease, and mutations in *SOD1* account for only ∼20% of familial and 2% of sporadic cases. A pathogenic expansion of the hexanucleotide repeat tract found in intron 1 of the *C9orf72* gene accounts for the largest proportion of ALS and frontotemporal dementia (FTD) cases. Two ASOs targeting *C9orf72* entered phase 1/2 clinical trials in 2021. BIIB078 (IONIS-C9Rx, Biogen & IONIS) was well tolerated but failed to meet efficacy endpoints.^173^ Similarly for Wave’s WVE-004 ASO which was also well tolerated—although it reduced levels of the pathogenic C9 repeat derived glycine-proline dipeptide repeat protein (poly(GP)) by 50% it resulted in no measurable benefit to clinical outcomes.^174^ Both were able to selectively lower *C9orf72* transcripts carrying pathogenic expansions in humanized mouse models in preclinical studies, but their development has now been ceased.

Another C9 ASO, afinersen, was able to lower poly(GP) up to 80% in mouse models by specifically targeting the expanded transcript, sparing an alternate transcript thought to splice out the expansion. Approval for compassionate use was received for a single patient where similar effects on the levels of poly(GP) in the CSF were seen.^175^ Ulefnersen (also known as jacifusen) is another MOE/phosphodiester/PS mixed backbone gapmer for ALS and FTD instead targeting the RNA encoding fused in sarcoma (FUS). Compassionate use approval was given to administer ulefnersen to a patient carrying the FUS^P525L^ mutation which results in an extremely aggressive juvenile onset ALS.^176^ Although progression was slowed, the recipient was in the late stages of disease when treatment started and died from ALS-related complication a year later. Postmortem showed a substantial decrease in burden of the pathological FUS-positive aggregates associated with FUS-ALS. Although exciting, it is difficult to draw robust conclusions from these single patient studies. Ionis began a phase 3 trial with ulefnersen in 2021. Further ASOs are being developed against targets whose lowering has prolonged survival in SOD1-ALS mouse models, including the p75 neutrophin receptor, acetylcholinesterase, and bone morphogenetic factor 4.^177^

Ionis Pharmaceuticals, in collaboration with Roche, developed tominersen, a nonselective stereorandom 2′-MOE gapmer ASO targeting HTT expression in HD. This ASO underwent a phase 1/2a clinical trial, an open-label extension study, and a subsequent phase 3 clinical trial. The phase 3 trial was ultimately halted due to poor safety signals and higher frequencies of adverse events in the higher tominersen exposure group without any improvements in clinical rating scoring.^178^ Dose-dependent increases in ventricular volume, increased leukocyte levels, and spiking neurofilament light chain levels in the CSF have been suggested to indicate either an inflammatory response, deleterious effects of lowering the wild-type HTT protein, or both.^178^ Hope may still remain for tominersen as it has returned to a phase 2 dose-finding trial with younger participants who may have responded better due to reduced disease burden. Wave Life Sciences also developed 2 stereopure allele-selective PS-ASOs for HD, WVE-120101 and WVE-120102 followed by the PS and phosphoryl guanidine (PN) backbone ASO WVE-003. Each target a different CAG-associated single nucleotide polymorphism (SNP) and can offer allele-specific lowering of mutant HTT to between 36% and 70% of the HD population. Both WVE PS-ASOs have been shelved as they failed to effectively lower mutant HTT and showed frequent adverse effects.^179^ The PS/PN-ASO WVE-003 has entered phase 1b/2a and has shown mutant HTT specific lowering in CSF. Wave expect to report more data in mid-2024.^180^ An alternative approach to treating HD not directly targeting HTT was being pursued by Triplet Therapeutics; however, they are no longer operational. They aimed to lower expression of the HD-onset modifier MSH3 in order to delay HD onset and progression driven by increased expansion of the pathogenic CAG repeat.^181^

Phase 1 trials are ongoing for the Biogen/Ionis ASOs BIIB094 and BIIB101 in PD, which target and degrade leucine-rich repeat kinase 2 (LRRK2) and SNCA transcripts, respectively. Mutations in LRRK2 result in a pathological increase in its kinase activity. Splice-switching ASOs targeting an exon key to LRRK2 kinase activity are also being investigated in preclinical mouse models with the aim to preserve the kinase independent functions of the protein.^182^ BIIB101 targeting SNCA is also currently in phase 1 trial in patients with multiple system atrophy, a rare aggressive synucleinopathy.^183^

Tau pathology occurs in several diseases either classed as a primary tauopathies where mutations are present or secondary where inclusions containing wild-type tau are present such as in FTD or AD. Therefore, tau-targeting ASOs could have broad application and Novartis are currently running a phase 1 trial of a tau ASO (NIO752) for the primary tauopathy progressive supranuclear palsy.^184^ Results from another phase 1b trials of an Ionis tau-targeting ASO (MAPT-Rx) were recently released showing no serious adverse effects and a dose-dependent lowering of total-tau levels in the CSF, down to 50%.^185^ This is the first ASO to the clinic for an AD relevant target, and the study was conducted in a small group of young participants with only mild AD. In addition to the proxy reductions in CSF tau species, reductions in tau positron emission tomography signal across the brain have also been shown, though whether these translate to therapeutically meaningful lowering will require evaluation in the larger phase 2 study currently underway.^186^

Therapeutic ASOs are also being pursued for a variety of channelopathies, including Dravet and Timothy syndromes. Dravet syndrome is a developmental and epileptic encephalopathy (DEE) that is most commonly associated with haploinsufficiency of the voltage-gated sodium channel alpha subunit Nav1.1 due to variants in the gene SCN1A.

Stoke Therapeutics is trialing STK-001 (zorevunersen), an ASO designed to compensate for the null SCN1A allele by upregulating productive expression from the unaffected wild-type allele. This occurs through exclusion of an exon in an alternate transcript from the unaffected allele that would otherwise be degraded by nonsense-mediated decay, increasing the levels of transcript encoding a functional channel subunit. This approach successfully reduced seizures and sudden unexpected death in epilepsy in Dravet mouse models,^187^^,^^188^ and has also been shown to rescue interneuron and cortical pyramidal neuron electrophysiological characteristics.^189^ Stoke therapeutics announced that STK-001 is “generally well tolerated” with “substantial and sustained reductions in convulsive seizure frequency” at the end of their phase 1/2a trial,^190^ and have received breakthrough therapy designation for STK-001 from the FDA as it moves toward phase 3 trials.^191^

DEEs can also arise due to gain-of-function mutations in ion channel genes, such as SCN2A or SCN8A, encoding the alpha subunits Nav1.2 and Nav1.6, and KCNT1 and KNa1.1. Gapmer ASOs designed to downregulate expression of these genes reduce seizures and extend lifespan in their respective gain-of-function mouse models.^192^, ^193^, ^194^ ASO-mediated lowering of these gene appears to reduce seizures in other mouse models of DEE such as Dravet, even where the targeted subunit is not a gain-of-function variant, suggesting a more generalizable approach to targeting ion channels may be possible across certain epilepsies.^195^^,^^196^ Phase 1/2 initial ascending dose studies are ongoing for an SCN2A targeting gapmer ASO (PRAX-222) from Praxis Precision Medicines in pediatric carriers of SCN2A gain-of-function variants.

Timothy syndrome is a multisystem channelopathy which occurs due to a gain-of-function missense variant in exon 8A of the CACNA1C gene encoding the *α*1 subunit of the voltage-gated calcium channel CaV1.2.^197^ Exon 8A is a developmentally enriched alternate splice isoform, and a recent approach to instead favor inclusion of the alternate counterpart exon 8 using splice-switching ASOs reduced disease-associated phenotypes in patient-derived cortical organoids and human-mouse in vivo grafts.^198^

Another neurogenetic disorder that is promisingly tractable using ASOs is Angelman syndrome, which occurs due to the loss of function of the neuronal ubiquitin-protein ligase E3A (UBE3A). This is most commonly due to deletions in the maternal UBE3A allele or larger deletions in chromosome 15.^199^ In neurons, inactivation of the maternal allele alone results in loss of function and disease as the paternal UBE3A allele is repressed due to imprinting. Repression of the paternal allele occurs through expression of the UBE3A antisense (UBE3A-AS) transcript derived from the distal portion of the oppositely orientated polycistronic small nucleolar RNA host gene 14 locus.^200^ Targeting UBE3A-AS expression improves neurological phenotypes in mouse models of Angelman syndrome by permitting expression of the paternal sense UBE3A allele.^201^ Gapmer ASOs have been developed to achieve this therapeutically by degrading the UBE3A-AS transcript, and have been shown to reactivate paternal UBE3A expression in mouse and NHP, with phenotypic rescue in mouse models.^202^^,^^203^ In a recent update, Ultragenyx has stated GTX-102, an ASO targeting UBE3A-AS, has shown positive effects on cognition, motor function, and communication with a consistent and acceptable safety profiles in phase 1/2 trials and are beginning phase 3 enrolment by end of 2024 (Ultragenyx, 2024). Ionis have reported similar positive phase 1/2 results and progression to phase 3 with their ION582 ASO, also designed to raise UBE3A levels by targeting UBE3A-AS.^204^ This is very promising for Angelman syndrome as a previous UBE3A-AS targeting ASO developed by Roche (rugonersen) had been indefinitely paused at phase 1 due to a lack of meaningful changes at endpoint.^205^

ASOs have also proven an attractive tool for personalized or N-of-1 treatment for rare disorders due to their flexibility in mechanism and target. Since the first example, the SSO milasen for a patient with neuronal ceroid lipofuscinosis 7 (a form of Batten’s disease), others such as atipeksen for ataxia-telangiectasia and jacifusen for FUS-ALS (as discussed above) have been developed.^206^ Increased accessibility and capacity for whole genome sequencing is making these types of individualized treatments viable within the lifetime of the patient.^140^

In addition to specific disease-gene lowering interventions, SSOs are also being used to target more general processes associated with neurodegenerative disease. Depletion of the RNA-binding protein TDP-43 from the nucleus and the associated proteinopathy is observed across multiple neurodegenerative diseases. Cytosolic aggregates positive for TDP-43 protein are observed in neurons in ∼97% of ALS cases, ∼45% of frontotemporal lobar degeneration cases, between 30% and 50% of patients with AD,^207^^,^^208^ and has also been described in other disorders including HD.^209^ TDP-43 plays a key role in mRNA processing and transport, and its loss results in aberrant splicing of these transcripts.^210^ One such transcript affected by loss of TDP-43 is that encoding stathmin-2 (STMN2), a protein involved in axonal regeneration. Baughn et al^211^ have recently shown that ASOs blocking a cryptic splice site that would normally be blocked by TDP-43 during health can rescue STMN2 expression in human and humanized mouse motor neurons. Restoration of STMN2 expression is also being pursued therapeutically,^212^ and QurAlis began phase 1 trials of a STMN2 SSO in 2023.^213^

Another such example includes stabilizing the expression of the neuroprotective protein RMB3, usually present only during hypothermia. The RMB3 mRNA constitutively contains an exon that leads to NMD of the transcript. This exon is excluded during splicing by a hypothermia-dependent mechanism allowing translation of the RMB3 protein. By using an ASO to skip this exon without hypothermia, Preußner et al^214^ were able to show promising neuroprotection in prion disease mice.

The varied effects that rationally designed ASOs can exert are ripe for combining to attack a therapeutic problem from multiple angles. For example, by combining the survival of motor neuron 2 (SMN2) SSO approach in patient fibroblasts with another ASO targeting the 5′-untranslated region of the *SMN2* gene, Winkelsas et al^215^ were able to upregulate SMN2 translation and increase levels of the functional SMN isoform in fibroblasts of patients with SMA patient. In an SMA mouse model, an attempt to recapitulate the protective effect of a mutation in the SMA-modifier gene *Chp1* by lowering it using an ASO in combination with an SMN SSO failed to impact progression.^216^ A similar approach combining the SMN SSO with an ASO targeting the SMA modifier *Ncald* showed more promising effects, reducing the loss of neuromuscular junctions and electrophysiological defects in SMA mice.^217^ As genetic modifiers and risk factors continue to be identified, more opportunities for combinatorial approaches will arise.

### D Novel chemistry and design

**Fig. 3:**
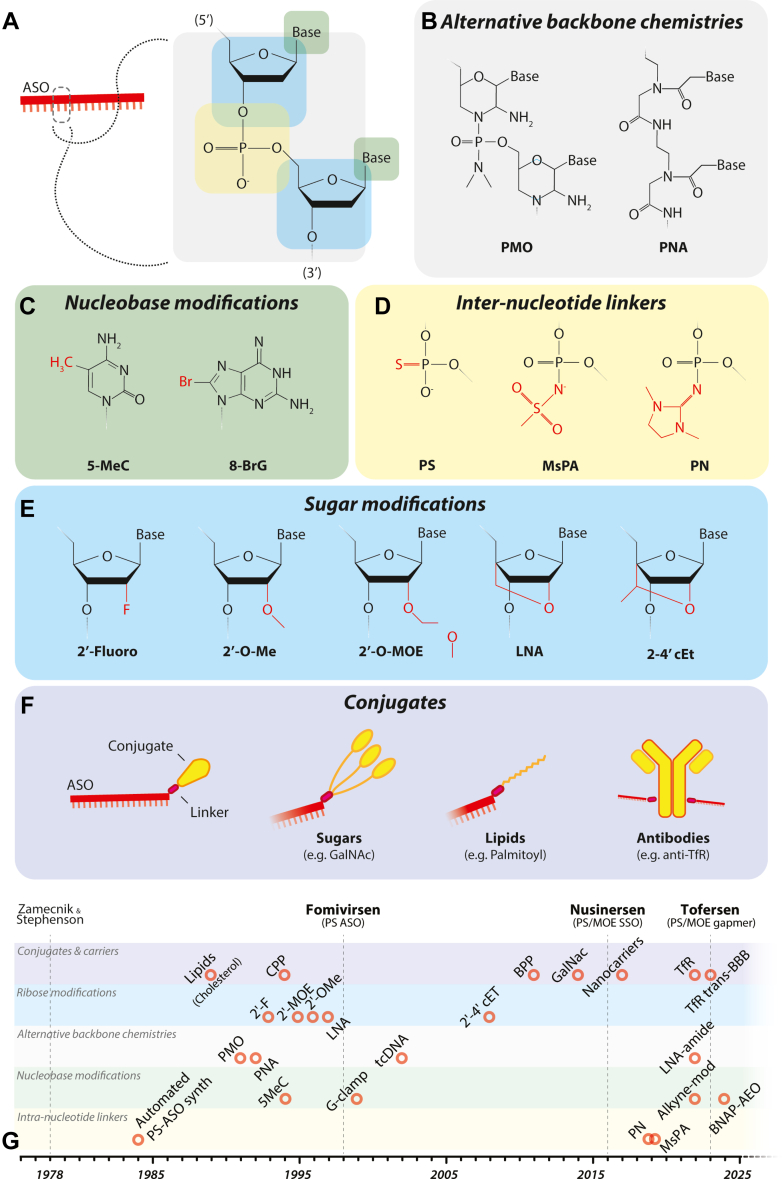
(A) The properties of short single-stranded oligonucleotides can be altered through chemistry modifications to create therapeutic ASOs. (B) This can be achieved through alternate backbone chemistries such as PMO, tricycloDNA (tcDNA), or peptide nucleic acids (PNAs); (C) use of nucleobase analogs such as 5-methylcytosine (5-MeC) and 8-bromoguanine (8-BrG); (D) changes to the internucleotide phosphodiester linker to PS, MsPA, or PN. (E) Further modifications can be made to the ribose ring most commonly to the 2′ position including 2′-fluorine groups, 2′ O-methyl (O-Me), 2′ O-MOE, and the use of bridged nucleic acids such as 2′-4′ LNA and 2′–4′ constrained O-ethyl (cEt). (F) ASOs can be conjugated to a wide range of additional factors to alter distribution and uptake. The position of the conjugate can affect activity, shown here attached to 3′ of an ASO by a variable linker. The triantennary GalNAc dramatically increase uptake of conjugated ASOs via asialoglycoprotein receptors on the cell membrane of liver hepatocytes. Lipid conjugates such as cholesterol and C16 palmitate have also been shown to improve tissue distribution. ASOs can also be linked to antibodies, F(ab) fragments, or nanobodies to specific targets such as the TfR. Delivery can also be altered by formulated of the ASOs into a variety of carriers such as LNPs (not shown). (G) So far only the PS-MOE ASOs nusinersen and tofersen have been approved for use for SMA and SOD1-ALS; however, since the first description of ASOs by Zamecnik and Stephenson^102^ in 1978 many novel chemistries, conjugates, and carriers have been characterized, suggesting the promise of next-generation ASO therapeutics is on the horizon.

A therapeutically useful ASO must be sufficiently resistant to degradation or excretion to reach disease-relevant tissues and cell types, be able to efficiently enter cells once there, transit to the correct compartment to encounter its target RNA, hybridize to its target sequence within that RNA, and finally invoke the intended effects upon that transcript. Each of these steps can by modulated by changes directly to the chemistry of ASO itself or by conjugation of biologically active ligands (Fig. 3). The extent to which an ASO can be modified depends on its intended function, whereas RNase H1–dependent ASOs must maintain a DNA core to ensure nucleolytic activity steric blocking ASOs can modified at any position (a mixmer) or completely modified as long as target affinity is maintained.

The classic phosphodiester bond found in DNA and RNA oligonucleotides is highly susceptible to degradation by nucleases. The first generation of ASOs made changes to the backbone to address this by using a PS linkage where sulfur replaces the nonbridging oxygen in the phosphate group of the phosphodiester backbone. PS backbones have increased resistance to nucleases, increased solubility, and increased half-life by slowing renal excretion through binding to plasma proteins.^218^

Although the PS linkage is the mainstay of current ASO designs, limitations to it use are becoming apparent such as increased in vivo toxicity. As such alternate linkages are being explored, including PN and mesyl phosphoramidate (MsPA) which both provide improved nuclease resistance over PS but with variable effects on potency.^219^^,^^220^ Current large-scale synthesis methods for PS-ASOs result in a mixture of stereoisomers with reproducible ratios, each of which can possess distinct pharmacokinetic/pharmacodynamic properties.^221^ Alternatives such as PN and MsPA linkages can be stereopure and the use of these at key positions in chimeric PS gapmers or SSOs improved delivery and potency in the mouse CNS, even compared with stereopure PS-ASOs.^222^^,^^223^ Wave used stereopure PN and PS chemistry in their *C9orf72* targeting ASO WVE-004 and showed target engagement in both mouse and human (though with no correlation with clinical changes, as discussed previously).^174^^,^^224^ Both PN and MsPA linkages are amenable to same solid-phase synthesis methods that have made the PS linkage so accessible.^225^ Modifications are also frequently made to the nucleobase and ribose ring of the nucleotides themselves. 5′-Methylcytosine is a common modification included to reduce immunogenicity.^226^ Further modifications include a “G-clamp” cytosine analog to increase target affinity,^227^ and more recently a wide panel of nucleobase modifications have been characterized.^228^^,^^229^

Modifications are also frequently made to the ribose ring of the nucleotides themselves. The most common modification found in current ASOs is the addition of a MOE group to the ribose ring. 2′-MOEs impose a more “RNA”-like conformation of the ribose which can enhance target affinity and specificity, as well as protect against nucleases.^230^ Alternate ribose modifications include 2′ O-methyl, locked nucleic acids (LNAs), and 2′-4′ constrained ethyl, though 2′-MOE remains the only sugar modification to be in an approved ASO. These modifications can be used throughout steric-ASOs and SSOs, or in key positions to form “mixmers.” As they are less similar to DNA in structure, they impair efficient RNase H1 activity, so restricting their use to a “gapmer” design with 5′ and 3′ modified wings flanking a core of 8–10 DNA nucleotides.^231^^,^^232^

Conventional ASOs simultaneously lower both mutant and wild-type mRNAs (as is the case for tominersen) potentially converting a dominant toxic gain-of-function effect to haploinsufficiency. The improvements to target affinity, understanding of enzyme mechanisms, and stability that these ASO modifications can yield enables the development of ASOs capable of discriminating SNPs. This can enable the targeting of a specific mutant allele where suitable SNPs or indels are present. This approach is desirable where the remaining wild-type encoded protein from the second allele could be compensating or maintaining baseline function, for example in the case of HTT in HD or C9orf72 in ALS.^233^^,^^234^

Another class of ASOs, PMOs, replace the ribose ring entirely with a morpholine ring and use phosphorodiamidate linkages between nucleotides resulting in an overall neutral charge. They possess increased nuclease resistance and reduced unintended protein interaction, making them less toxic at higher doses, though they are excreted faster and unable to recruit RNase H1 limiting them to steric blocking roles, such as in SMA or DMD.^235^^,^^236^ They are also currently more challenging than PS-ASOs to synthesize, though approaches to simplify large-scale synthesis may make them more accessible.^237^ Recent work by Baker et al^238^ aimed to synthesize reduced charge equivalents using LNA modified monomers with an achiral amide backbone compatible with existing solid-phase synthesis methods. These LNA-amide containing PS-ASOs showed improved gymnotic uptake and were effective at promoting exon skipping in cell culture.

### E Functionalization of antisense oligonucleotides through conjugation

Although ASO chemistry does contribute to tissue distribution and cellular uptake, conjugation to a second molecule or ligand has offered a new avenue both for getting ASOs to disease-relevant cells and into them. Lipids conjugates, such as palmitate and cholesterol, are able increase ASO uptake in cell culture and show stronger interactions with cell membranes than unconjugated ASOs.^239^ Lipid conjugates can also improve distribution of systemically delivered ASO to tissue such as liver, kidney, and bone marrow, but this does not necessarily improve uptake or target engagement.^240^ ASOs can also be formulated in nanocarriers such as LNPs to improve tissue distribution but while this has not yet proved as effective in the CNS as for other tissues there are promising results from technologies such as apolipoprotein nanodisk assemblies delivered systemically in mouse.^241^, ^242^, ^243^ Targeting disease-relevant cells or tissues is highly desirable in terms of minimizing off-target responses or toxicity in irrelevant cells—potentially reducing required dosage.

The use of receptor targeting ligands has proved very effective, and GalNAc conjugated ASOs (and siRNAs) have become the poster child for this approach. The GalNAc conjugate improves uptake by binding to asialoglycoprotein receptors on the cell surface of hepatocytes, leading to substantial increases in internalization and activity over unconjugated counterparts.^244^ Similar effects have also now been reported using the glucagon-like peptide 1 receptor agonist to target ASOs to pancreatic beta cells in mouse.^245^ Neuronal specificity has been shown to be possible in vivo using conjugates such as peptides, antibodies, or ligands that bind neuron-specific cell surface markers, such as neurotransmitter receptors.^246^ Nikan et al (2020)^246^ were able to enhance activity of an ASO in the mouse brain by conjugating it to neurotensin peptide, whereas Alarcón-Arís et al (2020)^247^ were able to lower *α*-syn in the monoamine neurons of PD mice and NHPs by conjugating ASOs to small molecule inhibitors of monoamine transporters.

To encounter their target RNA after uptake at the cell membrane, ASOs must escape the endolysosomal system. If they do not, they may be recycled back to the cell surface or trafficked to the lysosomes where they are degraded.^248^ It is possible to redirect ASOs to uptake and trafficking pathways that are more favorable for escape, but the method of escape remains poorly characterized and the majority of ASOs never leave the endosome.^249^ Even with the increased uptake seen with using GalNAc conjugates, only 1%–2% of ASOs escape the endolysosome system in cell models.^250^

Coadministration of endosomolytic small molecules or unconjugated peptides can improve intracellular ASO release.^251^^,^^252^ More direct release of the ASOs from the endosome can be mediated through conjugation to CPPs such as the HIV-1 derived Tat domain, though the underlying mechanism is still not well understood.^253^ CPP conjugates enhance delivery and improve the efficacy of both DMD and SMA SSOs in mouse at lower concentrations than unconjugated equivalents.^146^^,^^254^ Improved escape has also been reported for delivery using LNPs which are able to fuse with the endosome and evert their contents directly into the cytoplasm.^255^ Ligands are conjugated to the ASO by short variable linkers. Although noncleavable linkers ensure the conjugate cannot dissociate from the ASO, conditionally cleavable linkers can allow separation once the ASO is in a favorable position. For example, pH-sensitive linkers can be enzymatically cleaved only in the mildly acidic conditions of the endolysosomal compartment, reducing the potential for recycling back to the cell membrane with antibody or receptor ligand conjugates.^256^ Exon-skipping PMOs conjugated to TfR targeting fragment antigen-binding fragments with an endosomally cleavable linker have proved effective in DMD mouse models.^257^

Tailoring the ASO-conjugate linker and the conjugate itself will provide novel avenues for both improved cell-type targeting and more efficient entry into the cell. Conjugates that improve biodistribution and allow systemically administered ASOs to reach the CNS in therapeutically relevant concentrations are also emerging and are better discussed in the broader context of delivery.

### F Future directions in delivery of antisense oligonucleotides

Systemically delivered ASOs are primarily distributed to tissues such as the liver, kidney, and spleen, and while they are able to achieve sufficient therapeutic effect other tissues (eg, DMD ASOs such as eteplirsen in muscle) they require relatively high doses.^258^ This is complicated by the privileged nature of the CNS where the BBB prevents molecules larger than ∼400–600 Da from passing from the blood stream into the parenchyma.^259^ As ASOs are relatively large (∼5–10 kDa), hydrophilic, and negatively charged they are unable to pass the BBB, making systemic delivery difficult without modification. As a result, all current or under trial CNS-targeted ASO therapeutics are instead delivered locally by IT injection, or in the case of neuro-ophthalmological disorders, intravitreally.

Although brain regions more proximal to CSF flow are more readily accessible to IT administered ASOs, poor distribution to the deep structures of the brain continues to constrain the efficacy of current CNS delivery methods. This is an issue particularly in diseases where these structures may be areas of early degeneration and/or key to pathology, such as approaches for HD targeting mutant HTT expression in the striatum. ASOs delivered intrathecally to the CSF distribute throughout the neuroaxis, though it is not well understood how the processes that circulate fluid and molecules within the CNS impact ASO delivery. Tracing and distribution studies in vivo using ASOs targeting ubiquitously expressed targets have yielded insight into where IT-delivered ASOs end up in mouse and NHPs. Mazur et al (2019)^260^ found broad distribution with apparent faster accumulation in glial cells over neurons. The comprehensive distribution atlas generated by Jafar-Nejad et al (2020)^261^ showed similar broad distribution to, and activity in, each of the 35 regions assessed, and also highlighted a gradient of activity from superficial to deeper regions. Recent single cell analysis of ASO activity in the brains of rodents and NHPs by Mortberg et al (2023)^262^ has shown that the ASO activity observed at bulk levels is consistent at the single cell level, ie, uniform activity within the same cell types of a local region. This broad, region-uniform activity is of note in comparison to approaches such as viral delivery which may work effectively in the cells it transduces, but fail to transduce all cells.

These studies clearly show that IT delivery of ASO to the CNS can effectively reach a broad range of structures but with a bias toward superficial over deep areas. These effects are likely magnified in the human brain simply due to its substantially larger size compared with that of NHPs (macaques). ICV injection delivery is favored for difficult to reach structures such as the striatum even in rodent models, and has been similarly shown to be a more effective delivery route in sheep for siRNAs,^263^ and methods for repeated ICV dosing are being assessed in large animal models.^264^ Roche’s trials in HD with HTT targeting ASO tominersen used IT delivery, whereas the now defunct Triplet Therapeutics developed TTX-3360, an ASO which targeted the HD modifier gene MSH3 and was to be administered by ICV infusion via catheter.^265^

Overall, direct CNS delivery is highly invasive, prolonged, and more fraught with opportunity for drug-independent complications^266^^,^^267^ but is likely to remain a mainstay as other approaches are not mature. Intranasal delivery has shown promising results, with naked or cationic polymer-bound PS-ASOs being shown to effectively enter the CNS. ASOs conjugated to monoamine receptor binding ligands and repeatedly delivered intranasally were effectively taken up by monoaminergic nuclei in the substantia nigra of a PD mouse model,^247^ as were ASOs targeting HuR expression for neuropathic pain.^268^

Topical and direct deliveries can be limited by diffusion whereas the capillary network of the brain is estimated to cover 400 miles end to end,^269^ offering much more comprehensive coverage if the BBB can be navigated. This makes systemic delivery a highly desirable modality for ASO therapeutics, especially when combined with repeated systemic administration by intravenous or subcutaneous injection.

Postnatal maturation of the human BBB occurs between 3 and 6 months of age,^270^ beyond which further modifications of ASOs are required for efficient transit. Nusinersen for example does not effectively cross the BBB from either side.^271^ Systemic administration of either a PS-ASO or PMO targeting the same motif was able to affect SMN2 splicing in the CNS of neonatal mice,^236^ though neither were able to cross the BBB in mature animals. At the other end of age-of-onset spectrum, BBB dysfunction is a characteristic of both normal aging and is exacerbated in many neurodegenerative diseases so may make modeling ASO distribution from blood to brain more challenging.^272^ Efficient BBB penetration is essential if systemic delivery is to ever be a viable option for CNS targets, as is now being achieved by directed evolution approaches for AAV capsids.^273^^,^^274^

Drug agnostic nonsurgical methods to “permeabilize” the BBB could be applied to improve ASO transit, such as simple hypertonic substances, junction-disrupting peptides, angiogenic growth factors, or focused ultrasound, the latter of which gave favorable results recently, opening the BBB at multiple sites in a small AD group.^275^^,^^276^

Direct conjugation of ASOs to BBB-penetrant ligands has also shown promise in this area. The improved cellular uptake conferred by conjugation to CPPs discussed previously in terms of production uptake can aid BBB penetrance.^277^ Early iterations using derivations of arginine-rich CPPs such as the Pip6a-PMO SMN SSO have proven successful in mouse but are too immunogenic to translate.^146^ Recently, systemic delivery of an SMN PMO conjugated to the CPP DG9 has been shown to effectively distribute to peripheral and CNS tissue without significant immune response or long-term toxicity.^278^ These results are currently restricted to charge neutral SSOs for splice modulation. CPP conjugation of charged ASOs for RNase H1–dependent degradation has had mixed results.^279^

Similarly, conjugation to antibodies or ligands targeting surface proteins on the BBB can promote transit by transcytosis. The TfR is highly abundant on the surface of brain capillary endothelial cells. Systemic administration of an SMN SSO conjugated to novel antibody specific to murine TfR gave a good CNS distribution in an SMA mouse model and therapeutic levels of SMN2 splice modulation, though with a cell-type bias toward astrocytes, in the spinal cord at least.^280^ Small nanobody alternatives engineered for favorable binding kinetics to target TfR or other BBB surface proteins, such as TMEM30 and IGFR1, have also been reported. A recent pH-sensitive TfR nanobody showing improved uptake characteristics,^281^ as has an ASO conjugated to an engineered TfR-binding antibody Fc region.^282^ The independence from ASO chemistry in this approach may prove more flexible in comparison to CPPs.

In HD model mice, HTT-targeted ASOs conjugated to apolipoprotein A-I nanodisks reach the CNS when delivered intravenously and reportedly lower HTT expression in the striatum and cortex further than ICV delivery of the unconjugated ASO, and similar conjugates have shown good intranasal delivery previously.^243^^,^^283^ Encapsulation or complexing of ASOs with controlled compositions of lipid or polymer, or in cell-derived extracellular vesicles, can also increase BBB penetration. Functionalization of the encapsulation with various ligands, with similar rationales to the direct ASO conjugates described above, can further improve transit. For example, Min et al (2020)^284^ exploited abundant expression of the glucose transporter GLUT1 by encapsulating metastasis associated lung adenocarcinoma transcript 1 (MALAT1) ASOs in glucose-modified nanoparticles which were efficiently transcytoses through the BBB in fasted mice. Similarly in mouse, the addition of synthetic neurotransmitter-derived lipids to LNPs both allowed BBB transit and targeted delivery to neurons.^285^ A new generation of ASOs with minimally invasive delivery but maximally specific pharmacodynamics seems tantalizingly close but must be tempered by tolerance of unintended on- and off-target effects, and toxicity.

As touched upon earlier, improvements to efficacy and delivery must go hand in hand with improvements to toxicity—the lower doses required for higher potency ASOs with better on-target specificity and delivery could lead to improved toxicity profiles. Toxicity associated with different ASOs can arise from both sequence-dependent and independent factors. The predictable nature of the 15–20 Watson-Crick base pairs that can be formed by an ASO allows minimization of sequence-dependent off-targets by design.^286^ In silico predictions however require validation, and transcriptomics methods have shown ASOs can have significant effects on off-target transcripts with even single mismatches.^287^ As a result, these pipelines are being integrated into screens as both bulk or single cell transcriptomics and more rigorous bioinformatics methods become more accessible for triaging preclinical ASOs.

Hepatotoxicity associated with LNA containing gapmer ASOs has been linked to certain di- and trinucleotide motifs that result in off-target hybridization and degradation.^288^^,^^289^ Yoshida et al., (2022)^228^ have been able to reduce the effects of these motifs using novel nucleobase derivatives. Sequence-dependent toxicity can also occur independently of hybridization through other interactions with cellular proteins and changes to chemistry, such converting to 2′-constrained ethyl or PMO nucleobases, can reduce these interactions though at the cost of increased excretion.^290^ Additional chemistries and designs to mitigate sequence-dependent and independent toxicity without compromising stability and on-target affinity continue to be developed. One such method uses gapmer ASOs prehybridized to a peptide nucleic acid leaving a protruding toehold for strand displacement.^291^ As more data are collected from in vitro and in vivo applications of different ASOs, machine learning assisted approaches to ASO design may prove to be fruitful.^292^^,^^293^

Although ASO toxicity is primarily reported in terms of hepatotoxicity and general cytotoxicity, more efforts are now focusing on understanding the neurotoxic effects of ASOs in the CNS.^292^ Acute cytotoxicity and innate immune response activation have been reported in the CNS after ASO delivery, though some is surgery associated.^267^ PS-ASOs have been shown to activate toll-like receptors in a sequence-dependent manner, and TLR9 activation in the CNS has been shown to induce an inflammatory response.^294^^,^^295^ If CNS targeting ASOs make the switch to systemic delivery, this brings associated issues such as thrombocytopenia and additional hepatotoxicity.^296^ It seems more physiological models, such as human induced pluripotent stem cell derived neurons and other CNS cell types, could fill this gap and provide orthogonal validation (or exclusion) of ASOs at earlier stages. Three-dimensional organoid models for example have been shown to recapitulate and can allow ASOs to be trialed in human tissue–like structures ex vivo.

### G Conclusion

The ongoing preclinical development of ASOs, those already in clinical trials and the success of nusinersen and more recently tofersen, makes it seem likely a glut of ASOs may soon make it through approval to target a wide range of neurodegenerative disease in both adult and neonates. Equally, new chemistries and conjugates have made a move away from current invasive dosing modalities seem a possibility. ASOs, along with other nucleotide therapeutics, fill an important therapeutic niche, exemplified by their flexibility in mechanism of action and their relatively rapid design and implementation, which also makes them particularly amenable to N-of-1 trials. This expanding clinical pipeline holds promise for addressing the therapeutic needs of patients grappling with neurodegenerative diseases that were seemingly intractable only a short while ago.

## VI Gene editing tools for neurological diseases

### A Introduction

Gene editing stands as a promising molecular technology for precisely targeting and modifying the endogenous genome without the reliance on exogenous genetic materials.^297^ Gene editing involves the targeted DNA alteration, enabling the addition, deletion, or substitution of nucleotides. Gene editing techniques typically use specialized enzymes, such as CRISPR-Cas9, zinc finger (ZF) nucleases, or transcription activator-like effector nucleases, to introduce these modifications at desired genomic locations.^297^ Since the ground-breaking discovery of CRISPR/Cas9 in 2012, a plethora of novel gene editing tools have emerged and undergone preclinical testing, including for neurological diseases.^298^ Notably, a breakpoint moment arrived with the approval of the first-ever gene therapy using the CRISPR-Cas9 tool, a treatment for blood disorders such as sickle-cell disease and *β*-thalassemia, that deletes the faulty genes from patients’ stem cells (approved by FDA in December 2023).

Despite the current high costs associated with this treatment and the imperative need to assess its efficacy in clinical settings, this development has engendered a substantial sense of optimism within the scientific community, encouraging further exploration of gene editing modalities. Among the spectrum of gene editing tools available, CRISPR holds distinct promise due to its amenability to customization and adaptation for specific applications.^297^ Recent advancements in the evolution of the CRISPR/Cas9 system have created innovative technologies that in some cases circumvent the need for double-strand DNA breaks and DNA templates. CRISPR activators/inhibitors rely on defective Cas9 fused with transcriptional activator or inhibitor and have been used to upregulate haploinsufficient genes or could be used to decrease expression of gain-of-function mutations targeting the promoter regions.^299^ Similarly, epigenetic modulators act at the chromatin level to obtain the gene activation or inhibition.^300^^,^^301^ Base editing (BE) represents a transformative approach wherein a single target DNA base is directly and irreversibly converted into another, facilitated by a guide RNA and essential enzymes such as Cas9 and deaminase.^302^ Meanwhile, prime editing (PE) and its other recent evolutions, such as Programmable Addition via Site-specific Targeting Elements (PASTE),^303^ Phage-Assisted Continuous Evolution (PACE),^304^ and Prime-Assisted Site-Specific Integrase Gene Editing (PASSIGE),^305^ represent the way forward for future therapies, enabling targeted deletions, insertions, and all 12 possible base-to-base conversions.^306^

This breakthrough dramatically expands the scope of genome editing applications and holds the potential to correct up to 89% of known genetic variants implicated in human diseases. Over the past 5 years, PE has rapidly matured through versatile enhancements in architecture aimed at augmenting editing efficiency, targetability, and specificity. Moreover, leveraging previous accomplishments in the gene editing arena, PE has already been used to correct pathogenic mutations associated with genetic diseases both in vitro and in vivo.^307^ This trajectory underscores the profound potential of PE to boost the field of gene editing from laboratory experimentation to clinical implementation, offering renewed hope for patients with genetic disorders.^308^

### B Preclinical gene editing for neurological diseases

Gene editing is rapidly expanding into the realm of neurological diseases, offering promising avenues for treatment.^298^ Preclinical studies have targeted a range of common neurological disorders using gene editing techniques, encompassing AD, PD, HD, ALS, epilepsy, and autism spectrum disorder.

Gene silencing strategies have been used in most of preclinical work. For AD, efforts have focused on targeting mutations in the APP gene,^309^ modulating proinflammatory molecules like glia maturation factor,^310^ or reducing the secretion of amyloid-beta 42 by targeting Bace-1.^311^ For ALS, gene editing approaches have aimed to decrease the expression of SOD1.^312^^,^^313^ Similarly, in FTD, deletion of the C9orf72 promoter^314^ as well as the excision of the repeat expansion mutation^315^ has been explored. In HD, various strategies have been used to target the mutant HTT gene, including deletion approaches.^316^, ^317^, ^318^, ^319^, ^320^ Additionally, for Angelman syndrome, inhibition of Snord115 genes has shown promise for the reactivation of the silenced UBE3A locus.^321^

Lowering expression of pathological proteins has been achieved with ZF protein transcriptional factors. Tau gene MAPT and mutant HTT gene have been targeted, and their reduction rescued different pathological phenotypes in mouse models of AD^322^ and HD,^323^ respectively.

Gene activation using CRISPR activation has been investigated for channelopathies underlined by haploinsufficiency^324^ such as Dravet syndrome^325^^,^^326^ and Scn2a-related neurodevelopmental disease,^327^ for focal acquired epilepsy,^328^ as well as for obesity by targeting the *Sim* haploinsufficient gene in vivo.^329^ In vivo reprogramming of astrocytes into GABAergic neurons for PD has also been explored.^330^ Recently, ZF protein transcriptional factors, to upregulate gene expression, have been tested for Dravet syndrome, and they are in clinical trial.^331^

Epigenetic modifications have been studied for PD, involving methylation to downregulate the expression of the SNCA gene,^332^ and demethylation of the *MECP2* x-inactivation allele in Rett syndrome.^333^

Base editors have been used to modify a single base pair in the SMN2 gene converting it into a functional copy of SMN1 protein to treat SMA,^334^ and it has been shown that CRISPR BE can be successfully used in vivo to treat ALS,^335^ splitting the base editors with an intein-mediated transsplicing system, but the efficiency is still low.

Because PE is the most recently developed editing tool, it has been tested only for AD, with a study aiming to introduce protective mutations such as APOE3 R136S in vivo.^307^

Most of these approaches are still at the preclinical stage, but effort is in place to move them to the clinic soon.^298^ Interestingly, the first clinical trial for gene editing in neurological diseases has been designed based on ZF activators for Dravet syndrome (Encoded Therapeutics). The clinical trial is ongoing, and first patients have been injected intracerebroventricularly with an AAV9 carrying ZF activators for SCN1A driven by a specific inhibitory neuron promoter. These diverse approaches underscore the breadth of research underway to harness gene editing technologies for addressing the complex pathologies of neurological diseases.

### C Challenges in gene editing for neurological diseases

Although the conceptual framework of using gene editing for a myriad of brain disorders appears promising, several hurdles remain to be overcome before these techniques can be translated into clinical applications.^298^^,^^324^^,^^336^ Notably, neurons, the primary targets within the brain, pose a challenge due to their postmitotic nature, characterized by low levels of homology-directed repair mechanisms, and thus difficult for inserting DNA sequences.^337^ However, the advent of novel technologies such as base and prime editors has begun to address and ameliorate these obstacles. BE and PE represent significant breakthroughs, circumventing reliance on endogenous DNA repair mechanisms and enabling precise modifications at the single-base level or introduction of multiple bases.^308^ Prior to PE, approaches like homology-independent targeted integration^338^ or targeted knock-in with 2 guides^339^ offered a means to overcome neuronal accessibility challenges by leveraging non-homologous end joining repair for the insertion of DNA sequences. Although PE currently lacks the capability to insert large sequences, its advantage lies in improving clean insertions without generating indels, thereby enhancing gene editing efficiency.^308^ Nevertheless, very recently, an evolved PE tool, PASSIGE, has shown promising results in inserting very large DNA sequences, exceeding 10 kb, with a higher efficiency than previous PE approaches.^305^

A concern pertains to the risk associated with using double-strand breaks, whether for DNA insertion or indel generation, particularly regarding the predisposition of AAVs to integrate into the genome.^321^^,^^340^ This integration can potentially trigger unpredictable DNA rearrangements with potentially hazardous consequences for clinical translation. Moreover, the size constraints of commonly used viral vectors, such as AAVs (∼4.7 kb), pose a challenge in accommodating these gene editing tools.^341^

Furthermore, for gene editing nonmammalian proteins are used and another concern is the potential immunogenic response. Although the brain is an immuno-privileged organ, long-term studies of the potential immunological response to Cas9, for example, should be assessed carefully.^342^

Another critical concern revolves around the potential for off-target effects inherent in gene editing tools. Over the past year, considerable efforts have been dedicated to mitigating these off-target effects and enhancing the development of predictive algorithms as well as experimental detection and validation.^343^ By integrating computational predictions with experimental validation, researchers can better understand and mitigate the risk of off-target effects, ultimately improving the precision and reliability of gene editing technologies. Improving single guide RNA specificity and Cas9 variants, and the use of PE are ways to improve off-targets.^344^ However, it is noteworthy that when addressing brain disorders, detecting off-target effects presents a formidable challenge, particularly in scenarios of in vivo gene editing. Consequently, pre-emptively identifying off-target effects becomes impractical, underscoring the complexity of ensuring precision in brain-targeted gene editing.

### D Future perspective in gene editing for neurological diseases

AAV vectors remain the most widely used platform for CNS gene editing due to their neuron tropism and favorable safety profile. For delivery of large editor complexes, such as base or prime editors, dual or split AAV systems are being used to overcome the vector’s packaging capacity limitations, often using intein-mediated reconstitution or split-intein systems.^345^ However, considering the overall risks associated with AAV integration, size limitations of viral vectors, immunogenicity of the vector, off-target gene editing, potential for genomic integration, manufacturing cost, dose-limiting toxicity, and the imperative for transient expression—distinct from conventional gene therapy approaches—a shift toward nonviral transient methods emerges as a promising avenue for gene editing within the brain.^346^ These novel approaches include synthetic LNPs, formed by a cationic or ionizable lipid, a helper lipid, a polyethylene glycol lipid, and cholesterol. LNPs can encapsulate gene editing components and protect them during transit to the brain. LNPs offer advantages such as biocompatibility and much lower immunogenicity, customizable surface modifications for targeting specific cell types, and scalability for large-scale production. Although the use of LNPs in the brain is hampered by the natural accumulation in the liver when systemically administered, the developing of novel LNPs to target the brain is a pivotal goal for the field.^346^

Another emerging technology for delivering gene editing tools is virus-like particles (VLPs). VLPs are noninfectious assemblies of viral proteins and can package mRNAs, proteins, or ribonucleoproteins or viral genetic material.^347^ VLPs show efficient intracellular delivery, ability to encapsulate cargos, escape endosomes, and target different cell types. VLPs transiently deliver gene editing tools as mRNA or protein significantly reducing off-target gene editing and viral genome integration. For these reasons, VLPs are attractive delivering methods for gene editing.^346^ Other approaches include polymeric nanoparticles, CPPs, exosomes, or gold nanoparticle.^348^^,^^349^ These examples highlight the diverse range of nonviral delivery systems being investigated for gene editing in the brain, each offering unique advantages and potential applications in the development of novel therapies for neurological disorders.

Although the delivery field is very active around gene editing agents, a vast amount of research is investing in finding novel, shorter, and less immunogenic gene editing tools in an effort to overcome the current limitations and improve safety.^336^

As previously mentioned, the first-in-human clinical trial for a neurological disorder using gene editing is using ZF technology to upregulate gene expression in a rare haploinsufficiency condition—Dravet syndrome—through work led by Encoded Therapeutics. This milestone marks a significant advancement in the translation of gene editing technologies for brain disorders to the clinic.

However, the application of CRISPR-based systems to silence toxic gene expression—such as pathogenic proteins implicated in certain forms of dementia—may represent the next major step in the field. This approach holds particular promise for disorders characterized by gain-of-function mutations or aberrant gene regulation, where precise silencing could have a profound therapeutic impact.

Although more sophisticated platforms such as PE are still undergoing optimization—particularly regarding editing efficiency and the accurate prediction of off-target effects—their theoretical ability to precisely correct virtually any pathogenic mutation offers a compelling avenue for treating both rare monogenic and more common complex neurological diseases. As delivery methods and computational tools for target validation continue to evolve, these technologies are expected to become increasingly viable for clinical application in the coming years.

In recent years, the gene editing landscape has undergone significant expansion, drove by numerous research laboratories dedicated to advancing various facets of this technology with the ultimate goal of clinical translation.^298^ Notably, the emergence of several biotechnology companies has played a pivotal role in this endeavor, contributing to the development of novel Cas9 variants, innovative delivery systems, and refined methods for predicting off-target effects and reducing immunological response. This collective effort underlines a prevailing optimism regarding the imminent application of gene editing technologies in clinical settings, particularly in addressing the formidable challenge of neurological diseases. As such, the collaborative synergy between academic research and industry innovation promises well for realizing the transformative potential of gene editing in the realm of neurological therapeutics.

## VII Surgery for gene therapy

### A Introduction

The BBB is a complex neurovascular structure, highly conserved across species, that maintains homeostasis for water, ions, amino acids, hormones, neurotransmitters, and immune cells, and protects the brain from toxic and infectious agents. Although the density of the microcirculation within the brain parenchyma is high enough that no neuron is farther than 10–20 *μ*m from the nearest capillary, and has an estimated total surface area of 15–25 m^2^, small polar molecules such as amino acids and nucleotides, or molecules larger than 500 Da, will not penetrate the parenchyma from the capillaries.^350^ As only a small number of neurological diseases are currently treatable with small molecule drug therapy, the BBB represents a significant obstacle to therapeutic approaches to the brain. Most AAV vectors, commonly used for delivery of gene therapy, are 20 nm in size and therefore do not penetrate the BBB.

In addition to low transduction in the CNS, systemic administration of AAV-based gene therapy is challenging as it requires high doses, leads to high distribution to peripheral tissues, with potential off-target effects, and is inhibited by even low levels of circulating AAV-neutralizing antibodies, present in up to 32% of the population.^351^^,^^352^ Yet in some cases AAV9 therapy administered intravenously has been highly effective. Postmortem studies have shown widespread distribution and transduction of onasemnogene abeparvovec (Zolgensma) in the spinal cord and brain, with preferential uptake into the motor neurons in the ventral spinal cord in children with SMA.^353^ As the SMN protein is ubiquitously expressed, uptake and transduction into peripheral tissues is clinically beneficial. In most cases, however, alternative methods of delivery are required for gene therapy to reach their target in the CNS. Methods of agent delivery that have been investigated extensively and are now in use in clinical practice include delivery through the CSF pathways and direct injection into the brain parenchyma using CED. Techniques that have been used to temporarily open the BBB, such as high frequency ultrasound, have been trialed for chemotherapeutic agents for brain tumors, but have not yet been used clinically to deliver AAV-based gene therapy to the brain.

A recent comprehensive review has listed 35 AAV-based clinical trials currently underway for neurological disorders in adults and children.^7^ Of these, delivery is intraparenchymal in 8, in the cisterna magna in 8, into the CSF through the cerebral ventricles in 2 and through lumbar puncture in 9, and intravenously in 8. Clinical outcome data is awaited.

### B Cerebrospinal fluid delivery

Despite the rapid turnover of CSF, delivery of therapeutic agents through the CSF pathways has been an effective way to reach targets within the brain. This is an area of intense research, and over 4600 clinical trials have been conducted since 2005 on a variety of drugs and agents for various clinical indications, including oncological, such as leptomeningeal tumor dissemination, as well as gene therapy for neurological disorders for various clinical indications, including oncological, such as leptomeningeal tumor dissemination, as well as gene therapy for neurological disorders.^354^ The ependymal cells constitute the principal barrier between the CSF and the parenchyma, and as there are no tight junctions between them the concentration of a drug in the interstitial space is generally assumed to be equal to that in the CSF. There is, however, variable dependence on membrane transporters. CSF turnover results in an exponential decline in the concentration of drugs administered into the CSF over about 48 hours as they are diluted by bulk flow. Body position may also influence distribution between different CSF compartments.

Preclinical studies are critical to assess the translatability of intra-CSF delivery to neurological targets, and new therapeutic agents are typically first evaluated in rodents and larger animals prior to human trials to ensure satisfactory biodistribution.^354^ AAV serotypes vary not only in their intrinsic tropism, but also in their ability to transduce neurons in various locations in the CNS as well as in the outcomes they produce in different species.^355^ The large differences in brain and CSF volumes between species impact the kinetics and distribution of these agents. In addition, the turnover of CSF also varies; it is similar in most large animals, at 4 times per day in the human and monkey, equivalent to 350–600 *μ*L/min, but up to 13 times per day in rodents; this is presumed to be due to an increased heart rate.^356^^,^^357^ The fraction of CSF that circulates through the interstitial space is also variable, ranging from one–tenth in rats to one–half in humans.^354^ Pharmacokinetic measurements in such small volumes in animal models are challenging, and microdialysis methods have been effective at measuring drug concentrations within the interstitial space. Depending on their size, therapeutic agents move from the CSF along the perivascular spaces, in the same direction as arterial flow, driven by the pressure gradient caused by arterial pulsation, and pass through the interstitial space. This microcirculation within the brain, distinct from the bulk flow of CSF within the ventricles, the convexities of the hemispheres, and the spinal canal, has been referred to as the glymphatic system.

There are 3 routes by which gene therapy may be delivered into the CSF: through the lumbar theca (by lumbar puncture), through the brain ventricles (through a ventricular catheter or ventricular access device), and through the cisterna magna. The lumbar route is the simplest and can be used repeatedly with minimal morbidity. It is also the commonest route by which long-term continuous infusions into the CSF are carried out, through a surgically placed lumbar catheter. Agents injected into the lumbar theca however need to reach the ventricles and CSF compartments over the brain convexities, ascending along the spinal subarachnoid space against the bulk flow of CSF and CSF pulsations.^354^ Although AAV9 and AAV2.5 delivered through a single lumbar thecal bolus led to even transduction in the spinal cord and frontal cortex in NHPs, there remains concern that supratentorial distribution is generally poor and leakage out of the needle track at the site of injection further impacts delivery.^26^^,^^358^ The preservation of dural integrity is important, and a single dural puncture is superior to multiple attempts.^358^

Higher concentrations of therapeutic agents can be obtained by direct ventricular injection through a ventricular catheter or access device such as an Ommaya reservoir. In clinical practice, as gene therapy is only administered once, injection through a ventricular catheter, which is removed on completion of the injection, is preferable to implantation of a reservoir. NHP studies of injection of AAV with a gadolinium-based magnetic resonance (MR) contrast agent, using real-time MRI, combined with postinjection CSF sampling for AAV titers, have demonstrated satisfactory cortical exposure to AAV, particularly in the occipital and cerebellar regions, with this approach, as ventricular CSF flows out of the fourth ventricular outflow tracts toward the large arachnoid granulations along the posterior third of the superior sagittal sinus.^358^ However, penetration into deeper brain structures is not improved, and exposure is only cortical. In addition, this kinetic study demonstrated that spinal transduction is poor.

Combined distribution to both the brain and spinal cord has been demonstrated by injection into the CSF at the cisterna magna, through a suboccipital approach.^358^, ^359^, ^360^ This approach has led to widespread transduction in the brain and spinal cord in animal models.^26^^,^^358^^,^^361^ The most extensive distribution following injection into the cisterna magna was achieved when this approach was combined with additional injection at the lumbar level. Although potentially dangerous, cisterna magna injection has been carried out in small and large animals as well as in human trials. Injection into NHPs under fluoroscopic guidance, with gentle and continuous aspiration as the suboccipital membrane is punctured, increases the safety of the procedure.^359^ Another study demonstrated the use of a steerable soft-tipped catheter as used in neurointerventional procedures, and carried out by an experienced neurointerventionalist.^360^ The final position of the catheter, introduced through the lumbar route, was in the cisterna magna anterior to the pons; this was confirmed by coregistration of the intraoperative fluoroscopic image with the preintervention MRI scan. Additional agent was then introduced into the lumbar region as the catheter was withdrawn. Two children with Tay-Sachs disease were treated successfully by this approach. The NHP component of this study demonstrated maximal exposure to the therapeutic agent at the base of the brain and the spinal cord. Despite the close proximity of the anterior thalami and basal ganglia to the basal cistern, there was no penetration into these organs.^360^

### C Direct parenchymal delivery

Although injection of therapeutic agents into the CSF is useful when a wide exposure to the entire CNS is desirable, several other neurological diseases are compartmentalized, such as PD and AADCd, and it is then crucial to maximize transduction in a small and often deep part of the brain.

Direct injection of therapeutic agents into the brain parenchyma is carried out by CED and allows viral uptake and transduction in proximity to the point of injection. This route is particularly suitable for diseases that can be treated by expression of a gene in a particular location. In comparison with CSF routes lower doses of viral particles are required. Depending on neuronal connectivity, some viruses are able to disseminate to other areas of the brain by axonal transport, as occurs when AAV2-AADC viral genomes are delivered to midbrain dopaminergic neurons in AADCd.^99^ Different viral capsids allow anterograde or retrograde transport along interconnected circuits in a serotype-specific manner.^362^ Transport to diffuse cortical regions after CED to the thalamus, which acts as a central hub, has been demonstrated in NHPs^363^; Barua et al^364^ were subsequently able to demonstrate in their swine model that delivery of AAV vectors into the white matter leads to specific and effective distribution into the overlying cortex.

CED involves the bulk movement of a solute or drug along a pressure differential into the interstitial compartment, gradually replacing the extracellular fluid with infusate. The first injection studies, using blunt stainless steel 23-guage cannulae, were carried out in the corona radiata of anesthetized cats, using a large (transferrin) and a small (sucrose) molecule.^365^ These initial studies showed that “microinfusion” could effectively raise the concentration of a substance within the brain parenchyma to several orders of magnitude compared with that in the systemic circulation. Early trials using CED using diphtheria toxin for recurrent malignant gliomas demonstrated local tumor responses without systemic adverse effects.^366^

CED, driven by a pressure differential, can distribute an agent homogeneously throughout a high volume of interstitial brain tissue. The size of the agent is not a limiting factor, as the interstitial fluid is displaced by bulk flow of the solute containing the drug or viral particles. Distribution by this method is best for high molecular weight (>400 D) hydrophilic molecules or agents, which are therefore not easily cleared out of the interstitial fluid by absorption into the systemic circulation through the local capillaries.^367^ As a continuous pressure differential is required, it is essential that the materials used to inject the drug into the brain including the syringes, tubing, and implanted catheters are made of stiff noncompliant materials.

Several variables affect delivery of the therapeutic agent into the brain parenchyma. These include anatomy of the target site, infusion rate, infusion frequency, agent type, and concentration, as well as catheter design and placement.^367^ Delivery is defined by the ratio of the volume of brain permeated by the volume infused. This varies by the permeability of the target brain tissue; a higher ratio implies superior delivery. Brain gray matter (cortex) has a typical distribution ratio of 4:1, whereas white matter is more permeable and typically has a ratio of 7:1. The anatomy of the target region also defines the shape of permeation, as do the surface characteristics of viral particles.^368^ In the putamen, a frequent target in AADCd and PD, the presence of a rich network of lenticulostriate vessels around it may draw interstitial fluid and CED infusate into perivascular channels in a dorsoventral direction along a preferential extracellular flow pathway; a putaminal infusion approaching from a dorsal direction exploits this natural flow.^369^

Infusions are usually commenced at a low rate, starting from 0.1 to 5 *μ*L/min. The rate is then slowly increased over the infusion period. Effective infusion rates are specific to the catheter used and the target tissue. A trend to increase flow rates to reduce infusion time has identified the limitations of reflux and backflow, where the injected fluid is forced back along the tract of the cannula toward the surface of the brain. This reduces pressure at the point of injection, limiting effective distribution. Once an annular gap around the catheter is formed, and backflow is established, a new path with lower resistance is opened leading to loss of relatively large volumes of fluid.^370^ The needle tract effectively forms a pressure sink with lower hydraulic resistance than brain parenchyma.^371^ Rotational movement during insertion may compromise the parenchymal seal around the cannula and increase reflux. Reflux is more extensive when cannulae with large diameter are used.^372^ The position of the catheter tip with regard to the target region, as well as distance from the ventricle, the cortical surface, and major sulcal boundaries, also influence the extent of backflow and success of agent delivery.^373^

CED catheters in current use are up to about 32-guage in diameter. Mechanical disruption and trauma of brain tissue around the catheter caused during insertion, as well as the presence of air bubbles, intermittent blockage, pressure spikes during infusion, large catheter diameter, and catheter hardness, all increase the volume and extent of backflow.^371^^,^^374^ Insertion of a small soft catheter over a stylet increases the risk of introducing air bubbles. To limit this, catheters often have an outer coat that is more rigid and often made of silica obviating the need for an internal stylet.

**Fig. 4:**
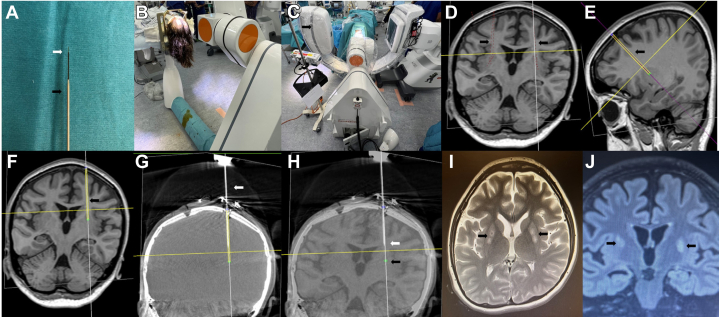
(A) Infusing end of the SmartFlow cannula, showing the termination of the rigid outer silicon cover (black arrow) and the stepped infusion tip (white arrow) which is designed to reduce backflow of infusate along the length of the cannula. (B, C) Operative room set-up for infusion of gene therapy into the putamen in a child with AADCd using the Renishaw robot. (B) The patient, supine, with the head immobilized to the robot in a Leksell stereotactic frame. (C) The patient and the robot arm are draped, and the mobile intraoperative 3-dimensional C-arm is positioned to allow intermittent imaging for verification of cannula trajectory. (D) T1-weighted MRI scan showing the right and left, anterior and posterior trajectories as planned on the NeuroInspire software (black arrows); the robot arm is aligned to the left anterior trajectory. (E, F) T1-weighted MRI scans in (E) sagittal and (F) trajectory planes (black arrows) to the left anterior putamen. (G) Intraoperative mobile computed tomography scan after insertion of SmartFlow cannula along the left anterior trajectory (white arrow); (H) when fused to the preoperative MRI scan, the trajectory of the cannula along the preplanned trajectory is verified (white arrow). The visible cannula falls short of the target (black arrow) as the length of the cannula beyond the silicon outer cover is radiolucent. (I, J) Intraoperative MRI scan after final injection and withdrawal of the cannula, (I) T2-weighted and (J) fluid-attenuated inversion recovery (FLAIR), confirming visible satisfactory infusion of therapeutic agent without coinfusion of gadolinium.

There are, currently, several devices used for CED. The most commonly used cannula is the SmartFlow Neuro Cannula (ClearPoint Neuro, California, USA), a step end-port catheter (Fig. 4A). A step, fashioned close to the tip of the cannula, reduces reflux up the catheter, increases perfusion and interstitial pressure around the tip, and improves distribution (Fig. 4). The first stepped catheter was composed of a 0.2-mm needle with a glued-in silica tubing, 0.168 mm in external diameter, that extended beyond the tip of the needle by 5–10 mm.^375^ Rigid cannulas, which contain ceramic or steel tubing with fused silica liners, are preferable for acute single event injection, as in the administration of gene therapy, as they minimize macromotion during implantation and injection.^376^

Renishaw PLC has subsequently developed a recessed submillimeter diameter catheter in which 2 guide tubes, an inner and a longer outer one, form a step 1.5-mm long just proximal (3–18 mm) to the catheter tip; the inner guide tube is shorter than the outer one, thereby forming a recess which, on insertion, is plugged with tissue and therefore limits further reflux.^370^^,^^377^ The developers argue that this cannula does not act as a point source of distribution, but rather as a controlled reflux device. A higher recess (or longer step length) led to a longer and narrower ellipsoid distribution; this was ideal for thalamic and putaminal delivery in a porcine model.^370^

### D Surgical techniques

Insertion of a CED catheter for gene therapy requires a stereotactic system to guarantee accuracy as well as a structure to support the position of the long cannula. Several such systems are available; they are mostly based on existing hardware and software used in routine neurosurgical practice to implant deep brain stimulation electrodes. Implantation of the Renishaw stepped catheter uses image guidance and stereotactic robot assistance, based on the NeuroInspire software (Renishaw PLC).^370^ Each component of the cannula is delivered over guide rods. The outer guide tube is delivered over a tungsten carbide delivery rod, just short of the implantation position. The inner guide tube is then passed over an inner steel rod under continuous aspiration to minimize entry of air into the tract. Finally, a 0.6-mm rod is advanced beyond the inner guide tube to the injection point, creating a preformed track for the unsupported flexible cannula. These catheters are inserted stereotactically using the Renishaw Neuromate robot, which may be used with or without a Leksell frame (Fig. 4, B–J). The Neurolocate stereotactic system (Renishaw PLC) does not require the head to be fixed in a Leksell frame, which has benefits for surgery in small children, under 2 years of age, where the soft calvarial bone can be penetrated or deformed, leading to loss of accuracy, by the Leksell pins. Although the accuracy of the Renishaw robot systems has been clearly demonstrated in tumor biopsies and stereotactic electroencephalogram procedures,^378^^,^^379^ the positioning of the cannula and the injection of the drug is not verifiable and is essentially blind. Use of an intraoperative computed tomography mitigates this disadvantage by demonstrating the proximal radio-opaque component of the cannula during insertion, and confirming a correct trajectory, but the radiolucent tip and its final position cannot be seen.

The ability to visualize the distribution of the drug in real time, so that appropriate corrective action can be taken, if necessary, during the infusion to ensure complete coverage of the target is useful. Even a small dural opening through a burr hole may cause leakage of CSF with entry of air and the possibility of some brain shift, introducing inaccuracy. One technique to achieve real-time CED, which therefore corrects for intraoperative brain shift, involves injection within the bore of an MRI scanner.^380^ The ClearPoint navigation platform (MRI Interventions) allows accurate drug delivery and real-time visualization in an intraoperative MRI setting.^380^, ^381^, ^382^ The SmartGrid (ClearPoint Neuro), a localizing adhesive grid, is positioned over the expected entry site before MR volumetric scanning, and informs the positioning of the SmartFrame, a skull-mounted frame which contains the infusion cannula guide. The ClearPoint software (ClearPoint Neuro) generates the trajectory and provides depth as well as coordinates on the XY axis. In addition, adjustments using hand controllers that extend beyond the bore of the magnet can be made by the neurosurgeon, allowing the expected error at the target to lie below 0.5 mm. A burr hole is then drilled through the mounted frame along the appropriate trajectory, and the SmartFlow cannula, after priming, is inserted to the required depth. Coinfusion with gadolinium allows real-time visualization of the injection; fast multiplanar T1 images are acquired every 5 minutes during the infusion. Once the infused fluid is seen in the target, the flow is increased as required by the protocol.^99^^,^^382^ Bilaterally mounted SmartFrames allow accurate simultaneous infusions in both hemispheres.

This system has been used widely, including for the delivery of AAV2, carrying a gene for AADC, into the putamen of patients with medically refractory PD. The primary benefit of the technique is that the volume infused may be varied depending on the coverage of the putamen, as it is visualized in real time.^380^

A further development that has been trialed in NHP studies involves a frameless skull-mounted ball-joint guide array.^383^ This device, made of polyether ether ketone (PEEK) and therefore MRI compatible, fixes to the skull through 3 screws; it rotates through 360° and has a maximum angulation of 16° to the vertical. Its center contains three 2-mm holes, each allowing cannulas, electrodes, or biopsy needles up to 16 gauge to be inserted through. The device also contains fiducials filled with gadolinium that allows registration using T1-weighted MRI scans. The software allows the trajectory of the cannula to be matched to the preplanned route. As in the ClearPoint system, real-time visualization of the infusion is also possible. The small size of the device is ideal for pediatric use, and allows multiple burr holes to be used simultaneously, either bilaterally or unilaterally with multiple directions to the same target. The 3 close parallel tracts allow real-time optimization of trajectory by switching to an adjacent port as a “rescue infusion.” Real-time adjustment in the MRI scanner also allows compensation for brain shift, related to loss of CSF or entry of air.^383^

Matching distribution of infusate to the target remains a challenge, particularly when the target is elongated or irregular and therefore difficult to cover with multiple spherical infusion points. The novel “infuse-as-you-go” technique has been described in a study that infused AAV solution to the putamen of NHPs through an occipital trajectory. The catheters were advanced in 2–4 mm increments during the infusion, under real-time MR guidance. Coverage of the putamen was superior to the standard transfrontal approach and could be achieved with a single trajectory. No reflux along the infusion cannula was noted.^369^

The development over the last 10 years of surgical platforms that reliably deliver gene therapy to the CNS, through the CSF or directly to parenchymal targets, has led to over 60 gene therapy trials. Fourteen of these studies involve intraparenchymal injection of AAV-based gene therapy in PD; other parenchymal or CSF routes are being used for patients with AADCd, AD, HD, and the MPSs.^362^^,^^384^ Further developments in understanding the impact of gene expression beyond the regions of injection, particularly in neurological disorders that have a broad impact on the CNS, and vector tropism along intact neural pathways, will be crucial to slowing down neurological deterioration and preserving function.

## Conflict of interest

Simon N. Waddington reports that consultancy fees for advisory services were paid to University College London Consultants, a wholly-owned subsidiary of University College London, from the following companies: Abingworth, Alchemab, Alnylam Pharmaceuticals, Atalanta Therapeutics, Catapult, Cure Ventures, Design Therapeutics, EQT, F. Hoffmann-La Roche, Ipsen, Iris Medicine, Latus Bio, LifeEdit Therapeutics, LoQus23, Novartis, Prime Global, PTC Therapeutics, Takeda Pharmaceuticals, uniQure, Vertex, and Wave Life Sciences Ltd. University College London Hospitals NHS Foundation Trust, Simon N. Waddington’s host clinical institution, received funding to run clinical trials for F. Hoffmann-La Roche, Novartis, PTC Therapeutics, and uniQure. Ahad A. Rahim and Simon N. Waddington are shareholders and receive funding from Bloomsbury Genetic Therapies Ltd. All other authors declare no conflicts of interest.
